# Alternative Transcription at Venom Genes and Its Role as a Complementary Mechanism for the Generation of Venom Complexity in the Common House Spider

**DOI:** 10.3389/fevo.2019.00085

**Published:** 2019-04

**Authors:** Robert A. Haney, Taylor Matte, FitzAnthony S. Forsyth, Jessica E. Garb

**Affiliations:** 1 Department of Biological Sciences, University of Massachusetts Lowell, Lowell, MA, United States; 2 Center for Regenerative Medicine, Boston University, Medical, Boston, MA, United States

**Keywords:** venom, transcriptome, toxins, *Parasteatoda*, alternative transcript

## Abstract

The complex composition of venom, a proteinaceous secretion used by diverse animal groups for predation or defense, is typically viewed as being driven by gene duplication in conjunction with positive selection, leading to large families of diversified toxins with selective venom gland expression. Yet, the production of alternative transcripts at venom genes is often overlooked as another potentially important process that could contribute proteins to venom, and requires comprehensive datasets integrating genome and transcriptome sequences together with proteomic characterization of venom to be fully documented. In the common house spider, *Parasteatoda tepidariorum*, we used RNA sequencing of four tissue types in conjunction with the sequenced genome to provide a comprehensive transcriptome annotation. We also used mass spectrometry to identify a minimum of 99 distinct proteins in *P tepidariorum* venom, including at least 33 latrotoxins, pore-forming neurotoxins shared with the confamilial black widow. We found that venom proteins are much more likely to come from multiple transcript genes, whose transcripts produced distinct protein sequences. The presence of multiple distinct proteins in venom from transcripts at individual genes was confirmed for eight loci by mass spectrometry, and is possible at 21 others. Alternative transcripts from the same gene, whether encoding or not encoding a protein found in venom, showed a range of expression patterns, but were not necessarily restricted to the venom gland. However, approximately half of venom protein encoding transcripts were found among the 1,318 transcripts with strongly venom gland biased expression. Our findings revealed an important role for alternative transcription in generating venom protein complexity and expanded the traditional model of venom evolution.

## INTRODUCTION

Venoms exhibit a high degree of biochemical complexity, with the venom of an individual organism being composed of up to hundreds of distinct proteins or peptides (Escoubas et al., 2006; King and Hardy, 2013). Such complexity may enable the rapid immobilization of a phylogenetically divergent array of prey and simultaneous disabling of many cellular functions (Olivera, 1997; Ushkaryov et al., 2004; Casewell et al., 2014). This complexity also makes venoms important resources for therapeutic drug discovery (Lewis and Garcia, 2003; Escoubas and King, 2009; Netirojjanakul and Miranda, 2017), targeted insecticide development (Schwartz et al., 2012; Windley et al., 2012; Smith et al., 2013), and neurophysiological research (Ushkaryov et al., 1992, 2004; Ashton, 2001; Sudhof, 2001; Deak et al., 2009; Silva et al., 2009).

The typical explanation for the generation of the diverse venom protein complement proposes a dominant role for gene duplication. After an initial gene duplication event, a shift to venom gland limited expression of the duplicate gene occurs. This is followed by further gene duplication events that generate diversity through the production of venom gland restricted toxin families with functionally distinct paralogs molded by the action of positive selection (Kordis and Gubensek, 2000; Fry et al., 2009; Wong and Belov, 2012; Schwager et al., 2017). Yet, other mechanisms such as the production of alternative transcripts expressed in the venom gland and producing venom toxins via alternative transcriptional start or polyadenylation sites, or through alternative splicing, could also generate protein diversity (Nilsen and Graveley, 2010; Pal et al., 2011; de Klerk and ‘t Hoen, 2015). Alternative transcription could thus act to enhance venom diversity, yet has been reported in only a small number of individual cases. For example, alternative splicing has been suggested to account for novel venom protein variants in snakes, lizards, and wasps (Siigur et al., 2001; Fry et al., 2010; Viala et al., 2015; Yan et al., 2017). However, the putative alternatively spliced variants in these studies were derived from *de novo* assemblies of short-read data or EST sequencing, and no genomic information was available to confirm whether these variants came from the same locus. Furthermore, while sequenced genomes, together with transcriptomic or proteomic datasets, are available for a limited set of venomous organisms (e.g., de Plater et al., 1995; Torres et al., 2000; Warren et al., 2008; Whittington et al., 2008, 2010; Kita et al., 2009; Sanggaard et al., 2014), no comprehensive exploration of the role of alternative transcription at venom genes or in venom composition has been undertaken in genome sequenced species.

In addition, the presence of alternative transcripts at venom genes may require a modified understanding of how an evolutionary shift in expression of a toxin transcript to the venom gland might be accomplished. If a gene that is duplicated has preexisting multiple transcripts, potentially only one of several alternative transcripts might shift expression to the venom gland, or such a shift to venom gland restricted expression could occur with no duplication from a gene that has multiple transcripts. In humans and other organisms, the use of alternative start or polyadenylation sites, or alternative splicing, play an important role in generating tissue-specific transcripts from existing genes (Farajzadeh et al., 2013; Hestand et al., 2015; Sanfilippo et al., 2017; Reyes and Huber, 2018). This process could provide a mechanism for altering venom composition through the generation of novel toxins expressed in the venom gland that circumvents the need for preceding gene duplication events.

Spiders are now amongst the venomous species with sequenced genomes (Sanggaard et al., 2014; Babb et al., 2017; Schwager et al., 2017), including the common house spider *Parasteatoda tepidariorum,* an emerging model for the study of arthropod development and evolution (Hilbrant et al., 2012). Although *P. tepidariorum* is a member of the spider Family Theridiidae along with the notorious black widows of the genus *Latrodectus,* its bites are generally not harmful to humans (Isbister and Gray, 2003; Isbister and White, 2004). Thus, this species is of importance for the study of venom evolution, as a contrast to black widow venom with its extreme toxicity to vertebrates. Additionally, a recent investigation of an earlier version of the *P. tepidariorum* genome assembly generated by the i5k (5,000 arthropod genomes) consortium focused on the diversity and evolution of two gene families whose proteins are abundant in black widow venom: latrotoxins and latrodectins (Gendreau et al., 2017), including a preliminary analysis of expression in a single venom gland RNA-Seq library. However, a full evaluation of the contribution of alternative transcriptional events at venom genes to venom diversity requires high-quality genomes together with replicated transcriptomes, as well as data on the protein composition of venom.

Here we integrated newly obtained data from multi-tissue RNA-Seq and tandem mass spectrometry (MS/MS) with an improved assembly of the recently sequenced *P. tepidariorum* genome (Schwager et al., 2017) to reconstruct transcripts at genomic loci, and characterize the complexity of this species venom by identifying the protein products of transcripts with MS/MS data from the secreted venom itself. In order to investigate the importance of alternative transcription in generating venom diversity, we tested whether alternative transcripts are characteristic of genes encoding venom proteins, and also whether alternative transcripts from the same locus may generate multiple venom toxins with distinct protein sequences or architectures. We also explored the expression patterns across tissues of alternative transcripts found at venom genes. In particular, we tested whether the expression of venom protein encoding transcripts is restricted to, or highest in, the venom gland, as predicted in a traditional model of venom evolution (Fry et al., 2009), and as expected given that their products are presumed to be secreted toxins. We also tested whether venom genes have all transcripts, or only a subset, that are venom gland restricted, or whether some transcripts at venom genes are primarily expressed outside of the venom gland, suggesting that they may have alternative functions.

## MATERIALS AND METHODS

### Laboratory Protocols for RNA-Sequencing

Adult female *P. tepidariorum* were maintained in the laboratory on a diet of crickets, and were fed 3 days prior to being placed under 5 min of CO_2_ anesthesia for extraction of venom glands, silk glands, and ovaries and isolation of the cephalothorax after venom gland removal. Extraction of RNA was performed with a modified Trizol protocol, followed by column purification using an RNeasy kit. As it was necessary to pool venom glands from 11 to 12 individuals to obtain sufficient material for RNA extraction, and to average out inter-individual variability, we also combined silk glands, ovaries and cephalothoraxes from 3 individuals in each replicate. Two replicates per tissue type, for a total of eight biologically replicated RNA-Seq libraries were generated, with each replicate from a different pool of individuals.

### Transcript Reconstruction and Expression Analysis

Quality control and library preparation for Illumina sequencing were performed at the Deep Sequencing Core Laboratory of the University of Massachusetts Medical Center. All cDNA libraries were sequenced using a 100 bp paired-end protocol on an Illumina HiSeq 4000. Low-quality sequence was trimmed from reads at a Phred score threshold of 20 and Illumina adapters were removed with TrimGalore 0.3.7 (https://github.com/FelixKrueger/TrimGalore) incorporating FastQC 0.11.3 (http://www.bioinformatics.babraham.ac.uk/projects/fastqc/) to verify read quality: median quality scores were at least 30 across all bases in all reads. Trimmed reads were aligned to the *P. tepidariorum* genome assembly (NCBI Accession: A0MJ00000000.2; Schwager et al., 2017) using Hisat2 2.0.5 (Kim et al., 2015) with settings for downstream transcriptome assembly, a maximum sequence mismatch penalty of 4 and a minimum mismatch penalty of 1. To generate a more comprehensive reference annotation incorporating expression information from isolated tissue libraries, alignments were performed for each of the eight libraries from this study, two libraries (venom gland and silk gland) from a previous study (Gendreau et al., 2017) and a single library from ovary tissue produced by the i5k project (i5KConsortium, 2013). Stringtie 1.2.4 (Pertea et al., 2015) was then used to assemble transcripts using an existing annotation for the Schwager et al. assembly as a reference in each run. Individual assemblies from each library were merged with the reference genome annotation for *P. tepidariorum* to produce a final annotation, which was used for estimation of transcript abundance. All transcripts in the final merged annotation were identified by comparison to the *nr* database using the blastx command in Diamond 0.8.23 (Buchfink et al., 2014) using the best local alignment at an e-value cutoff of 1e-5. The final merged annotation was compared to the reference annotation to identify novel genes expressed from previously intergenic regions (class code “u”) using Cuffcompare in the Cufflinks suite (Roberts et al., 2011).

Analysis of differential expression was performed using the R package EBSeq (Leng et al., 2013). Read counts for use in EBSeq were generated using the prepDE script provided with Stringtie and five counts were added to each value to prevent underflow errors during calculations in R when counts were zero in both replicates for a tissue. We performed pairwise comparisons between venom gland and the three other tissues and identified transcripts with expression significantly upregulated in the venom gland at a false discovery rate of 5% in each comparison. The intersection of the three sets constituted the final set of venom gland upregulated transcripts. To confirm novel splice junctions and to calculate junction support as reads mapped to junctions present in the merged final annotation, we used STAR 2.5.0a (Dobin et al., 2013) using default parameters.

### Protein Identification and Sequence Analysis

Milked venom was obtained from SpiderPharm (Yarnell, AZ) where it was produced by pooling venom extracted by electrostimulation from ∼300 anesthetized adult female *P. tepidariorum*, in order to generate sufficient material and to capture the broadest sample of venom components. Lyophilized venom was separated on polyacrylamide gels and divided into 8 fractions for subsequent analysis, including a small peptide fraction. Each fraction was trypsin digested, and separated on a Thermo/Proxeon nano-HPLC apparatus. Eluted proteins were subjected to mass spectrometry using a Linear Trap Quadropole (LTQ) Velos Orbitrap tandem mass spectrometer at the University of Arizona Cancer Center Proteomics Shared Resource. Three technical replicates of this analysis were performed. A database for searching with mass spectra was produced from predicted proteins from all transcripts in the final merged annotation of the *P. tepidariorum* genome assembly. While polymorphism is present in the RNA-Seq reads, database transcripts were each represented by a single consensus sequence derived from the genome assembly that summarizes mappings from reads across all libraries. Proteins were predicted first by translating all open reading frames >90 nucleotides for each transcript and then using an in-house Perl script to choose the longest protein in the frame of the best blastx hit, or the longest protein in the absence of a BLAST hit. Sets of transcripts with 100% identical predicted proteins were identified using CD-hit (Li and Godzik, 2006) after the trimming of protein predictions to the first methionine residue. Protein domain predictions were performed with InterProScan (Quevillon et al., 2005). Predictions of protein toxicity were performed with Clantox (Naamati et al., 2009), and of inhibitory cystine knot structural folds with Knoter1d (Gracy et al., 2007).

Spectra were matched to proteins in the reference database using Sequest version 1.3.0.339 and the results were viewed in Scaffold 4.4.7. To consider a protein as present in the venom, peptide probabilities were set at 95% and protein probabilities at 99%, with a minimum of 2 peptide matches per protein required. The decoy false discovery rate for these settings was 0%. Proteins with shared peptides that could not be discriminated were placed into groups, while proteins that were not supported by independent evidence were deemed not present in venom. The identified protein list was purged of common contaminants (keratin, trypsin) and of a likely arthropod contaminant (hemocyanin).

## RESULTS

### RNA-Seq Based Transcript Prediction Helps Reveals Venom Composition

Novel genes and transcripts were predicted from the *P. tepidariorum* genome assembly using eight new RNA-Seq libraries, as well as three previously published libraries (Gendreau et al., 2017), and merged with an existing annotation for this genome (Schwager et al., 2017). This resulted in a final tally of 58158 genes and 90093 transcripts (Supplementary File 1), of which 23989 novel genes and 25214 novel transcripts were defined as expressed regions of the genome that did not overlap with any existing annotated gene. However, only 4,718 (19.7%) of these putative novel genes had a BLAST hit to the *nr* database at an e-value of 1e-5 or better, a far lower proportion than that for the total number of genes with a BLAST hit in the entire genome (73.6%), and the function of the remaining expressed regions remains unknown.

The SDS-PAGE gels of *P. tepidariorium* venom showed multiple protein bands, including high molecular weight bands in the size range of latrotoxins (110–130 kDa), as well as a number of smaller bands, with the smallest in the 10–15 kDa range (Figure S1, Figure S2). After discarding contaminants, we identified via tandem mass spectrometry a minimum of 99 proteins across all gel slices in the venom of the common house spider (Table S1). Fifty-three proteins were unique identifications: a single protein that could be discriminated from other proteins in the database by the peptides identified in the experiment. However, 46 identifications were of a group of proteins that were not distinguishable by the peptide data. Further, 26 of these 46 groups contained 2–5 proteins that were distinct in sequence when compared from the putative start codon, yielding a potential maximum of 139 distinct proteins in the venom. The remaining 20 groups contained only identical proteins, but these proteins had been included in the database as they were produced by distinct transcripts.

**FIGURE 1 |:**
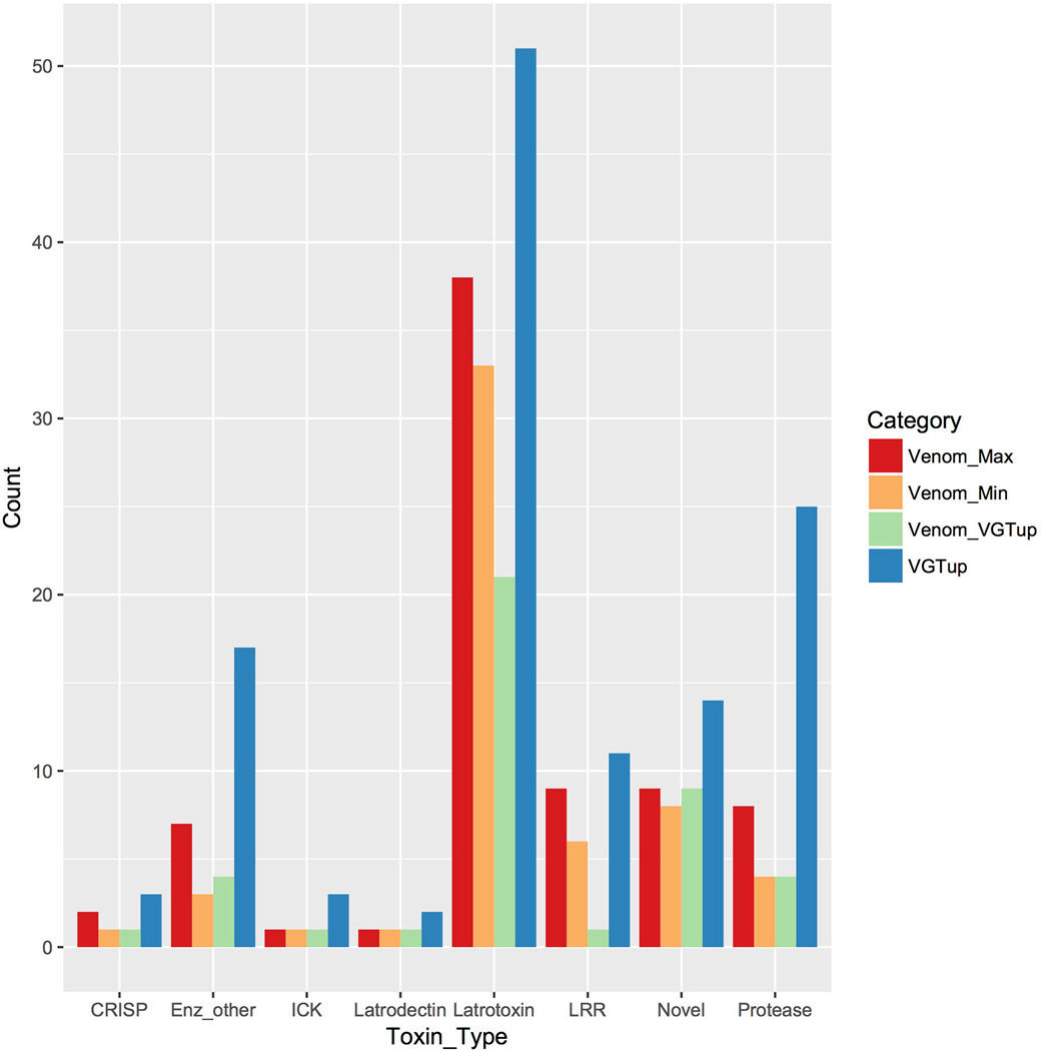
Known or putative toxins and spreading factors found in secreted venom (red, maximum number including proteins indistinguishable by MS; orange, minimum number) or upregulated in the venom gland (blue). Overlap between presence in the venom and venom gland upregulation is shown in green. *CRISP,* cysteine-rich secretory protein; *Enz_other,* hyaluronidase, amylase, lipase; *ICK,* inhibitory cysteine knot toxin; *LRR,* leucine-rich repeat protein; *protease,* serine and metallo-protease; *Novel,* putative toxin family identified in Gendreau et al. (2017).

For enumeration of types of venom components, we provide a conservative minimum estimate assuming that only a single predicted protein from a group contributed an individual identification (99 total proteins), and a maximum estimate that includes all proteins from each group (139 total) with distinct sequence, but that could not be discriminated. Proteins were assigned to toxin or other categories of interest using the BLASTx hit with lowest e-value for the transcript from which they were predicted. Latrotoxins were the most numerous type of known toxin in *P. tepidariorum* venom, with 33–38 different latrotoxin proteins present. However, latrodectins (small accessory proteins to latrotoxins), inhibitory cysteine knot toxins (ICKs) and cysteine-rich secretory proteins (CRISP) were not diverse in venom, with only 1–2 representatives each (Table 1; Figure 1; Table S1). There were 3–7 proteases, and 3–7 proteins corresponding to other enzymes (lipases, amylases, and hyaluronidases) in the venom. We identified 6–9 proteins with homology to leucine-rich repeat proteins (Table 1; Figure 1; Table S1), which were also identified in confamilial black widow venom (Haney et al., 2014). There were 24–34 distinct proteins in venom with a best BLAST hit to uncharacterized proteins (Table 1), including 8–9 proteins derived from genes in a novel family identified by Gendreau et al. (2017) with transcripts having very high expression in *P. tepidariorum* venom glands, and 2–3 proteins lacking a BLAST hit. Of these 26–37 distinct uncharacterized or unknown proteins, 14–17 were <200 amino acids in length, and rich in cysteines (6–14 Cys residues). However, zero were predicted to have an inhibitory cystine knot structural motif, although 1 was predicted as a probable toxin and 3 were labeled as possible toxins by the Clantox server (Table S1).

### Venom Genes Typically Have Multiple Transcripts and Encode Distinct Proteins

In sum, 86 genes encoded at least one protein identified in venom, 28 of which produced only a single transcript (of which 15 were latrotoxins), while 58 were multiple transcript genes. The proportion of multiple transcript genes (58/86: 67.4%) contributing to venom was significantly higher by chi-square goodness-of-fit test (χ^2^ = 115.82, df = 1, *p <* 0.0001) than the proportion of genes across the genome that encoded multiple transcripts (11922/58158: 20.5%). Identified events producing alternative transcripts at venom multiple transcript genes included alternative 5^/^ splice sites (7, 6.6%), alternative 3^/^ splice sites (3, 2.8%), exon skipping (13, 12.3%), mutually exclusive exons (26, 24.5%), alternative first exons (27, 25.5%), and alternative last exons (30, 28.3%). However, no venom gene alternative transcripts were produced through intron retention. Accounting for the presence of grouped and indistinguishable proteins, including identical proteins produced by distinct transcripts, there were 169 transcripts from these genes encoding the maximum 139 distinct venom proteins. Most of these transcripts (141, 83.4%) were encoded by the 58 multiple transcript genes (Table S2), and a slight majority (72) of these 141 transcripts were novel to this study. Together with the 141 transcripts encoding a venom protein, the 58 multiple transcript venom genes had an additional 135 predicted transcripts, for a total of 276.

These 276 transcripts produced 210 different protein sequences, and individual genes encoded 1–22 distinct variants. Forty-four of the 58 multiple transcript genes had transcripts that encoded more than one distinct protein, accounting for 196 of the 210 (Table S2: column 2). The greatest number of distinct proteins encoded at a locus occurred at genes with BLAST homology to chitinases (MSTRG.35296, 27 transcripts, 22 distinct proteins), endothelin-converting enzymes (MSTRG.35640, 22 transcripts, 20 distinct proteins), and latrotoxins (MSTRG.45528, 23 transcripts, 17 distinct proteins).

**FIGURE 2 |:**
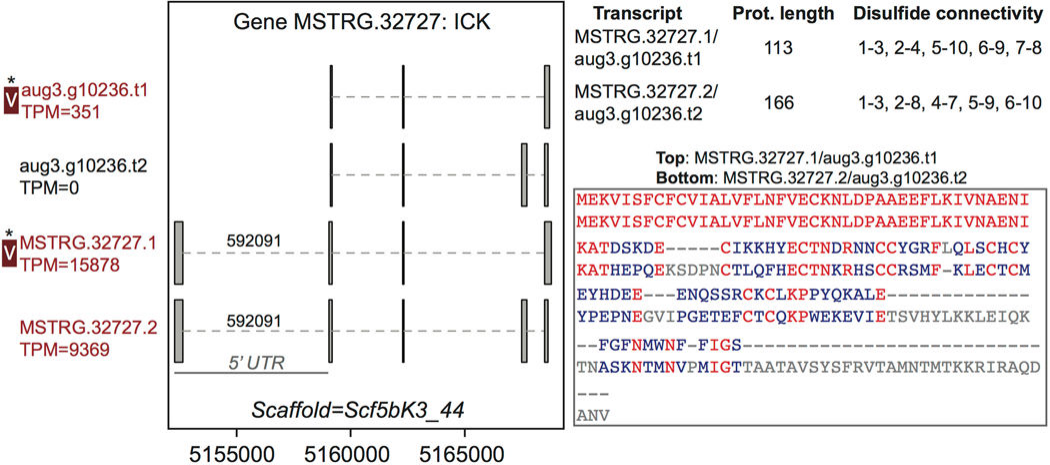
A venom protein-encoding multiple transcript gene produces two distinct proteins, but only one is identified in venom. Locus MSTRG.32727 has homology to inhibitory cystine knot toxins, and has four transcripts which produce two different predicted proteins. For each of the two distinct proteins, two different transcripts encode the same protein sequence, which vary by the presence or absence of an intron bearing 5’ UTR. The proteins vary in length and are divergent in sequence, with identical residues in red. Each has five predicted disulfide bonds, but the predicted pattern of connectivity is different. Transcripts labeled “V” have protein products identified in venom by MS. A “*” indicates that these proteins cannot be discriminated by the MS data. Numbers over introns indicate the number of spliced reads supporting novel junctions across all libraries. Also shown is venom gland expression in TPM rounded to the nearest whole number, and whether the transcript is upregulated in the venom gland (dark red text) relative to other tissues.

Among the 44 multiple transcript genes producing multiple protein variants via alternative transcripts, peptide evidence from the venom MS experiment was sufficient to distinguish a single protein as present in venom for 15 (Table S2, column 3). For example, gene MSTRG.32727, an inhibitory cystine knot toxin, produced four transcripts, two of which were novel to this study and which both possessed a 5 untranslated region (UTR) bearing a highly supported intron with canonical splice sites not present in the previous genome annotation (Figure 2; Supplementary File 1; Table S3). These four transcripts produced two divergent predicted proteins (each encoded by two distinct transcripts) that differed in predicted disulfide binding pattern, an important determinant of function in ICK toxins. For this gene, however, only one of these proteins was distinguished in venom (Figure 2) by unique peptide matches.

### Venom Genes Can Contribute More Than One Protein to Venom

**FIGURE 3 |:**
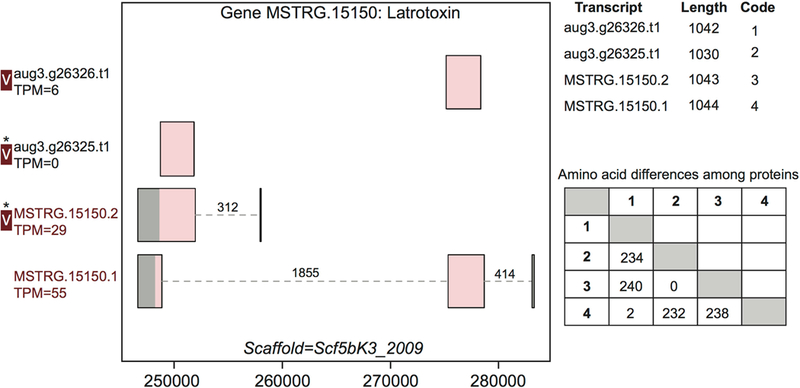
A venom protein-encoding multiple transcript gene produces two venom proteins. Locus MSTRG.15150 has homology to latrotoxins, and has four transcripts (two novel to this study), which produce four different predicted proteins, which vary in UTR length and exon-intron structure. Coding regions are shaded pink, and non-coding gray. The proteins produced vary slightly in length but also in primary sequence, as enumerated in the lower table on the right, in which numbers correspond to transcript codes in the upper table. Transcripts labeled “V” have protein products identified in venom by MS. A “*” indicates that these proteins cannot be discriminated by the MS data. Numbers over introns indicate the number of spliced reads supporting novel junctions across all libraries. Also shown is venom gland expression in TPM rounded to the nearest whole number, and whether the transcript is upregulated in the venom gland (dark red text) relative to other tissues.

**FIGURE 4 |:**
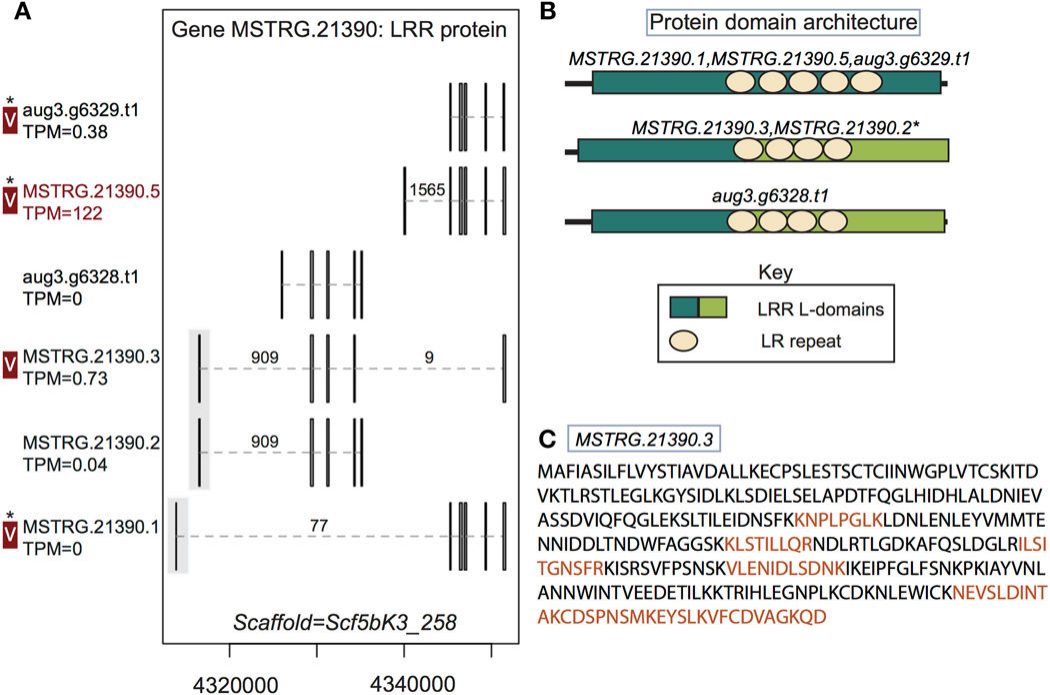
A multiple transcript gene encodes two structurally distinct proteins found in venom. **(A)** Locus MSTRG.21390, with homology to leucine-rich repeat proteins, has 6 distinct transcripts, four which are novel to this study, encoding 5 distinct proteins. Novel upstream exons containing coding sequence together with a short untranslated region are shaded in gray. Transcripts labeled “V” have protein products identified in venom by MS. A “*” indicates that these proteins cannot be discriminated by the MS data. Numbers over introns indicate the number of spliced reads supporting novel junctions across all libraries. Also shown is venom gland expression in TPM rounded to the nearest whole number, and whether the transcript is upregulated in the venom gland (dark red text) relative to other tissues. **(B)** Three different protein domain architectures were predicted from protein primary sequences at this locus, and the first two shown were identified in venom. The leucine-rich repeat locations and size are identical for MSTRG.21390.3 and MSTRG.21390.2, but LRR L-domain start and end positions differ by 1 basepair, so these sequences are considered to have the same architecture. **(C)** Sequence of the novel protein from transcript MSTRG.21390.3 indicating peptides mapped to the sequence from MS data (orange).

In contrast, eight of the 29 other venom multiple transcript genes producing multiple proteins contributed at least two distinguishable proteins each to the venom (Table 2, Table S2: column 4) as assessed by the MS data, accounting for 23 total distinct venom proteins (16.5% of all distinct proteins). Each of the 8 genes were from regions of the genome with more than one paralogous gene in the existing annotation of the dovetail genome assembly (Schwager et al., 2017). However, expression data in this study indicated more complex transcriptional patterns in these regions, with novel UTRs, transcriptional start sites (TSS), introns and exons defining new transcriptional units (Supplementary File 1; Figure 3, Figure 4, Figures S3-S8). For these eight genes, and for venom multiple transcript genes in general, novel transcripts that yielded a protein in venom generally had strong support, including numerous reads spliced across novel exon-exon junctions (Table S3).

For example, gene MSTRG.15150, a latrotoxin, had four transcripts in our updated annotation, two of which were present in the previous annotation, and which were annotated as separate genes lacking UTRs (Figure 3). The two novel transcripts from this study show novel TSS and UTRs with three previously undetected introns. These introns possessed canonical splice sites and were highly supported by spliced reads (Figure 3; Table S3). Each of the four transcripts produced a unique protein. One transcript novel to this study, MSTRG.15150.1, encompassed the entire region, and combined segments of two distinct coding regions to produce a novel protein sequence. The other, MSTRG.15150.2, produced a longer predicted coding sequence distinct from transcript aug3.g26325.t1, which it subsumed, via an upstream start codon. At least two, and possibly three, proteins predicted from this locus were identified in venom (Figure 3). The protein from transcript aug3.g26326.t1 was identified, and while the peptide data indicated that the protein produced by transcript MSTRG.15150.1 was not in the venom, the proteins produced by MSTRG.15150.2 and aug3.g26325.t1 could not be discriminated, and hence one or both may be present.

### Distinct Venom Proteins From Novel Transcripts Are Supported by MS Data

In two cases, transcripts novel to this study received support from the MS data. First, a leucine-rich repeat protein gene, MSTRG.21390, contributed at least two distinct proteins to venom. This locus had six transcripts (four novel to this study), which in total produced five distinct proteins (three novel to this study). Again, these novel transcripts included UTRs not present in the original annotation, as well as novel exons and introns with canonical splice sites, which were generally highly supported by spliced reads (Figure 4; Table S3; Supplementary File 1). Three of these novel exons added coding sequence at the 5^/^ end of the transcript (Figure 4). At least two, and possibly three of the proteins produced, with different arrangements of leucine-rich repeats, were found in venom (Figure 4B), including the novel protein predicted from transcript MSTRG.21390.3, which spanned genes from the original annotation, combining exons into a novel combination, and was uniquely identified by MS data. Novel transcript MSTRG.21390.1 produces a protein distinct from one encoded by two different transcripts (MSTRG.21390.5 and aug3.g6329.t1) due to 7 amino acids predicted from a novel upstream exon. However, the two proteins are otherwise identical and cannot be discriminated by peptides in the current MS data set, and hence one or both may be present in venom in addition to the protein from MSTRG.21390.3.

A second novel predicted protein sequence, from the chitinase gene MSTRG.35296, was uniquely identified in venom. This protein was one of two distinct proteins from this locus that were identified in venom, and was encoded by two novel transcripts (Figure S3) that spanned and shared exons with three genes in the original annotation (Figure S3;Supplementary File 1). Although this locus was transcriptionally complex, with 27 transcripts (Figure S3; Supplementary File 1) and 22 proteins, for the most part novel introns, which contributed to the generation of protein variation, were highly supported by spliced reads and possessed canonical splice sites (Table S3).

### Alternative Transcripts May Further Contribute to Venom Protein Diversity

An additional 21 (of 29) genes produced from 2 to 9 different proteins, of which a minimum of 1 and a maximum of 4 (Table S2, column 3) per gene were potentially present in venom. However, although the potential venom proteins were distinct in sequence, they were only matched by shared peptides. In sum, these 21 genes may contribute a minimum of 21 and a maximum of 63 proteins to the venom. Gene MSTRG.6569 illustrates this result, having four transcripts (two novel) that produce four distinct proteins that cannot be differentiated by peptides derived from the MS experiment (Figure S9), in addition to four other novel transcripts that do not produce a venom-identified protein. As in previous examples, novel transcripts were generally highly supported by overall read counts and by spliced reads across novel exon-exon junctions (Table S3). This phenomenon was also observed among the eight genes contributing more than one protein to venom. Five of these genes (MSTRG.25517, MSTRG.15150, MSTRG.21390, MSTRG.2390, MSTRG.32970) also produce additional distinct proteins that may be present in venom, but could not be differentiated by the MS data, as only shared peptides were matched. Thus, up to seven additional distinct venom proteins may be produced by these loci.

### Venom Gene Transcripts Show Variable Spatial Expression Patterns

Reads from eight sequenced libraries from venom glands, silk glands, ovaries, and cephalothorax (with venom glands removed) were mapped to the genome (Table S4), and used to identify 1,318 venom gland upregulated transcripts (VGTup), transcripts with a pattern of expression highly biased toward the venom gland (Table S5), which came from 1,095 genes. This is a substantially higher number than the 355 genes with a VGTup found in a previous study (Gendreau et al., 2017) using an earlier genome assembly, although 108 (30.4%) of the genes found to have a VGTup in the previous study had a VGTup in the current analysis, and 448 of the 1,095 were novel genes defined in this study as expressed regions of the new genome assembly. Overall, the 1,318 transcripts that constitute the venom gland upregulated set had an enhanced likelihood of producing a protein in the secreted venom [1.5% of transcripts in genome, 47.3% (80 of 169) of all transcripts with predicted proteins in venom]. Yet, more than half of all venom proteins were produced by transcripts that were not venom gland upregulated, and instead displayed a range of expression patterns across tissues, and surprisingly included transcripts with zero venom gland expression.

This pattern of expression was observed at both single and multiple transcript venom protein encoding genes. Of the 28 single transcript genes that encoded a protein identified in venom (Table S6), only 14 (50%) had transcripts that were significantly upregulated in venom gland. These 14 transcripts, including 4 latrotoxins (Table S6), were among the most highly expressed transcripts in venom gland, with 11 in the top 1% of venom gland expression rank, and all 14 in the top 5% (average TPM = 278.3), although several showed some level of expression in other tissues (Table S6). However, the remaining 14 transcripts, although also producing venom proteins, mostly exhibited low expression across tissues (Table S6), including the venom gland (average TPM = 0.6), although 11 were latrotoxins. Furthermore, six of these transcripts had no measurable expression in venom gland (TPM = 0).

**FIGURE 5 |:**
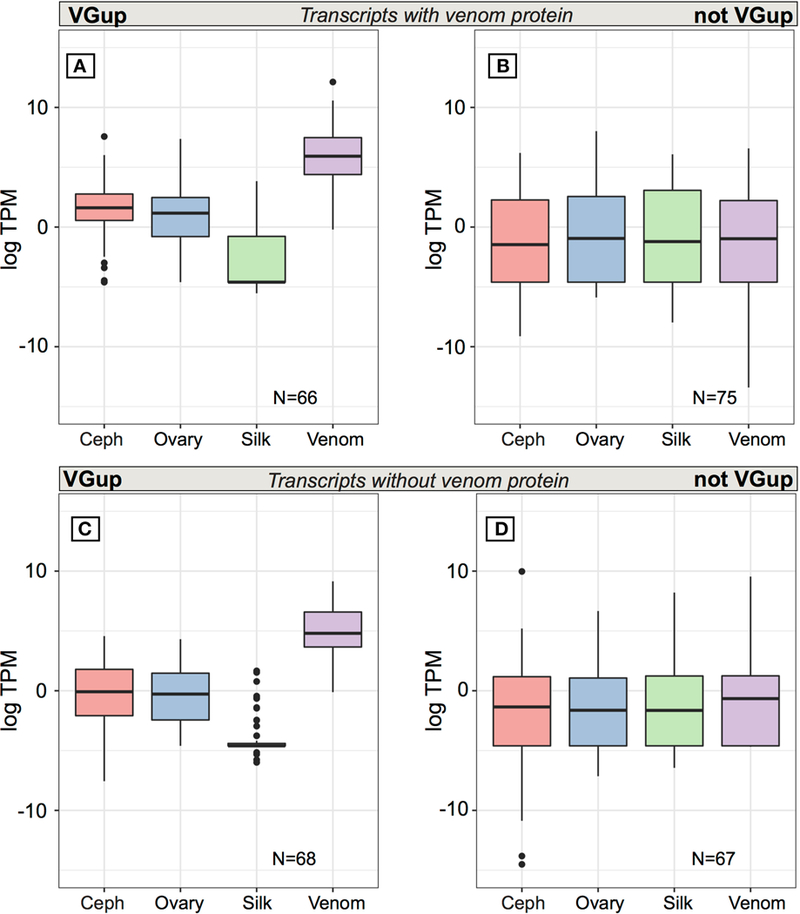
Average expression (TPM) across tissues for transcripts from multiple transcript genes encoding at least one protein identified in venom. Top panels are for transcripts encoding a venom identified protein while the bottom panels show the range of values for transcripts from the same set of loci whose encoded proteins could not be identified in venom. Panels **(A,C)** show values for transcripts that were significantly upregulated in venom gland relative to the other three tissues, while panels **(B,D)** show values for transcripts that were not significantly upregulated in venom gland. Black bars show median values, while the interquartile range is indicated by the boundaries of the colored boxes. Whiskers are 1.5× the interquartile range (IQR) and dots indicate outliers beyond 1.5 × the IQR.

At multiple transcript venom genes, less than half of all venom protein-encoding transcripts exhibited venom gland upregulation (66 of 141: 46.8%) and high abundance in the venom gland (Table S6; Figure 5A), with 56 transcripts in the top 1% of venom gland expression and 63 of 66 in the top 5% (average TPM = 4697.6), including 18 latrotoxins (Table S6). Yet, as with single transcript venom genes, 75 other transcripts from venom multiple transcript genes that encoded a venom protein were not venom gland upregulated, and were generally lowly expressed in all tissues, with median TPM < 1 (Figure 5B). Furthermore, 27 had zero expression in venom gland (Table S6), including eight latrotoxin transcripts. However, 29 (37.2%) of these 75 transcripts that do not exhibit venom gland upregulation produced a protein that belongs to a group of venom proteins indiscriminable by our MS data (Table S1, Table S6), in which at least one other transcript is venom gland upregulated. Hence, it is possible in these cases that the venom gland upregulated transcript actually produces the protein found in venom. Also, 12 of these 75 transcripts, while not significantly upregulated, do have their highest average expression in the venom gland (Table S6). Surprisingly, however, nine of these transcripts that were not grouped at protein level with a VGTup actually showed relatively substantial expression in all tissues, and 30 had higher average expression in at least one other tissue than in venom gland.

Multiple transcript genes that contribute a protein to venom also had from 0 to 23 transcripts whose encoded protein was excluded from being present in venom by the current MS data (Table S2), which in theory could be transcripts that have an alternative function to that of a toxin. Yet, the pattern of expression of these transcripts is similar to those that do produce a venom protein. Approximately half (68 of 135: 50.4%) of these additional transcripts at venom protein encoding loci are significantly upregulated and abundant in the venom gland (Table S6; Figure 5C), with 48 in the top 1% of venom gland expression and 65 in the top 5%. The 67 remaining transcripts at these genes, not significantly upregulated in venom gland, are generally lowly expressed (<1 TPM) on average in all tissues (Table S6; Figure 5D), and although 13 had highest average expression in the venom gland, 22 transcripts had 0 TPM in the venom gland. Five of these transcripts showed expression > 5 TPM in all tissues, and 41 had higher average expression in at least one other tissue than in venom gland. Three had zero or very low expression in the venom gland, and expression in at least one other tissue that was at least an order of magnitude greater.

## DISCUSSION

### Common House Spider Venom Is a Diverse Mixture

Animal venoms are complex, heterogeneous solutions, and often contain a diverse suite of molecules (Casewell et al., 2013). We found that common house spider venom contains a variety of proteins, a finding similar to that from other spiders and from more distantly related organisms whose venom composition has been assayed (Liao et al., 2007; Yuan et al., 2007; Duan et al., 2008, 2013; Tang et al., 2010; Palagi et al., 2013; Colinet et al., 2014; Undheim et al., 2014; Brinkman et al., 2015; Himaya et al., 2015; Borges et al., 2016). Proteins identified include homologs of ICKs, CRISPs, and enzymes, including proteases, lipases, chitinases, amylases and hyaluronidases, which may act as spreading factors or be modified as toxins, in addition to other components. However, as in the confamilial *Latrodectus hesperus* (Haney et al., 2014), latrotoxins, atypically large neurotoxins that act on the presynaptic membrane (Grishin, 1998), were the most diverse group found in *P. tepidariorum* venom. While previous functional characterization of four sequenced latrotoxin molecules (Rohou et al., 2007) indicated targeting to specific groups (mammals, crustaceans, insects), knowledge of functional variation among the expanding set of sequenced latrotoxins is lacking. Also lacking is an understanding of how these large neurotoxins functionally interact with smaller toxin molecules, including ICKs and CRISPs, present in theridiid venom.

### Alternative Transcripts and Venom Diversity

The presence of products from numerous toxin genes of the same type in *P. tepidariorum* venom is consistent with the standard notion of gene duplication as a predominant force in the diversification of venom toxins (Kordis and Gubensek, 2000; Fry et al., 2009; Wong and Belov, 2012; Gacesa et al., 2015). Yet, proteomic diversity can be generated by other mechanisms, and distinct toxins that might vary in mechanism or prey specificity could also be produced by an alternative transcript, arising from alternative start or polyadenylation sites, or by alternative splicing (Graveley, 2001; Maniatis and Tasic, 2002). Our results using an integrative venomics approach (Calvete, 2017) suggest that alternative transcripts may have a role in producing venom complexity, and that multiple mechanisms were involved, with the exception of intron retention. While previous studies have suggested that a few specific toxin variants may be derived from alternative transcripts (Siigur et al., 2001; Fry et al., 2010; Viala et al., 2015; Yan et al., 2017), alternative transcription may in fact be prevalent at genes producing venom proteins, as genes that encoded proteins found in venom were much more likely to produce multiple transcripts than the genomic background rate. These alternative transcripts generated numerous distinct predicted protein sequences, creating a diverse pool of protein variants at individual loci that produce venom proteins.

The involvement of alternative transcripts in the generation of venom protein diversity were most clearly demonstrated by eight genes defined in the new annotation developed for this study that had more than one protein unambiguously identified in venom by mass spectrometry, as these genes together accounted for 23 distinct venom proteins. However, in the pre-existing genome annotation, these regions contained multiple distinct paralogous genes (Schwager et al., 2017). Yet, data from this study indicated that these genes were bridged by highly supported novel transcripts that produced distinct proteins, and these transcripts had generally high levels of read support, including numerous reads spliced across novel exon-exon junctions. Furthermore, support from independent venom proteomic data was found for two novel transcripts from two different genes (MSTRG.21390, a leucine-rich repeat protein, and MSTRG.35296, a chitinase) where the novel protein predicted from region-spanning transcripts, which combined exons from multiple previously annotated genes, was identified. Thus, while it seems likely that the original annotation did not capture the full complexity of the transcriptome, and that the deep sequencing of tissues in this study has revealed more complex multi-exon genes, additional confirmation from proteomic data that these predicted gene-spanning transcripts are not artifacts and thus yield venom proteins, is needed. In addition, in these genomic regions the processes of gene duplication and alternative transcription do not appear independent, and may interact to produce variation, as proteins of a similar length are produced from transcripts spanning one part, or all, of the region (Figures 3, 4, Figures S3-S8), and more than one complete coding region appeared to be present. This phenomenon appears similar to that documented in human cells, where transcripts have been identified that contain segments from two adjacent genes (Akiva, 2005; Frenkel-Morgenstern et al., 2012), have been labeled “chimeric” RNAs. However, it has recently been argued that this transcriptional pattern is not unexpected, and that transcribed regions may simply be genes requiring re-annotation (He et al., 2018).

Furthermore, the contribution from protein sequence variants at venom multiple transcript genes may be more extensive than those identified from these eight genes. For most venom multiple transcript genes, a level of ambiguity remained as to which of several proteins occurred in the venom, as the only peptides identified as present in the venom by MS are shared among related proteins. Hence, while these genes may contribute only a single protein to venom, the possibility remains that some are actually contributing more than one distinct protein and adding to venom complexity, and in general the transcripts producing protein variants are highly supported by RNA-Seq. The MS data is restricted to a single experiment, and the augmentation of the available proteomic data might aid in resolving this ambiguity, and in confirming whether additional proteins from these genes, including those at low levels, are present in common house spider venom.

### Many Venom Gland Selective Transcripts Are Not in Venom

An expectation of the traditional model of venom evolution is that a switch to selective venom gland expression of a new toxin gene occurs subsequent to its origination via duplication from a non-venom “body” protein gene that fulfills some typical physiological role (Kordis and Gubensek, 2000; Fry et al., 2009; Hargreaves et al., 2014). We identified a complement of transcripts with expression that was significantly biased toward the venom gland (VGTup) as a proxy for this selective expression, as in other theridiid spider species (Haney et al., 2014, 2016). Yet, while this set of venom gland upregulated transcripts were more likely than expected to produce a protein found in venom, most did not, and many actually occur at genes that do not produce any venom protein. These upregulated transcripts could be required for specific cellular functions in the venom glands, and do not act as venom toxins. Alternatively, and particularly for transcripts that are also homologous to known spider toxins, such as latrotoxins and ICKs, this finding could represent post-transcriptional controls acting in venom gland tissue, which prevent the protein products of transcripts at these genes from appearing in venom. Post-transcriptional processes may also explain why distinct proteins from VGTup at other venom protein encoding loci were not found in venom, although protein products from other transcripts at the same locus were. Given their selective expression in the venom gland, this suggests that the presence or absence in venom of the products of these transcripts could be under control at the level of translation or secretion, and could provide a reservoir of additional toxin variants. Post-transcriptional processes have been put forth as an explanation for the discrepancy in transcript and protein abundance in snakes (Casewell et al., 2014), although this did not lead to the absence of the products of highly expressed venom gland transcripts in the venom, as in *P. tepidariorum,* suggesting the possibility of differences in levels of post-transcriptional control among phyla.

### Transcripts at Venom Genes Are Not Always Expressed Selectively in Venom Gland

In contrast with the selective expression in the venom gland as posited in the traditional model of venom evolution (Fry et al., 2009), many of the individual transcripts that produce venom proteins in this study have some level of expression in other tissues (silk, ovary, or cephalothorax). Given that protein products found in venom are likely to be toxins, the expression of their encoding transcripts in other tissues could indicate a defensive function. For example, toxins have been identified in confamilal *Latrodectus tredecimguttatus* eggs, where they may serve to protect from predation (Yan and Wang, 2015). Alternatively, it is conceivable that these transcripts do not yield a translated product in other tissues, an hypothesis that could be tested with proteomic data. The presence of alternative transcripts at venom genes also adds an additional layer of complexity to the pattern of expression expected from the traditional model, which involves selective expression in the venom gland at the level of the gene, which presumably encodes a single transcript. However, venom protein genes are biased toward multiple transcripts, and have transcripts that do not produce products in venom, or show venom gland restricted expression. In fact, a subset show low or no venom gland expression, but do show substantial levels of expression outside the venom gland, and three showed venom gland expression at or near zero, with much higher expression in other tissues. This pattern of variable expression could indicate that in certain cases transcription in the venom gland is achieved at the level of individual transcripts, whether by switching of expression to the venom gland during evolution for a single transcript at a multi-transcript gene, or by generation of alternative transcripts at a gene whose expression has shifted to the venom gland and reversion to expression in other tissues, with functions in other tissues mediated by alternative transcripts as opposed to by paralogs. This topic merits further study, particularly with regards to the gene regulatory mechanisms that might be involved, and how they may evolve, which requires multi-species data on alternative transcription.

However, interpretation of patterns of transcript expression is complicated by the potential influence of environmental conditions on gene expression, and venom gland expression of some transcripts could potentially be enhanced by certain environmental stimuli, including exposure to different prey communities (Daltry et al., 1996; Binford, 2001; Sanz et al., 2006; Gibbs et al., 2011), and hence the full complexity of a venom can only be exposed by assaying venom from populations across a range of environments. This is supported by data from cone snails, which indicates that venom composition can vary in response to environmental stimuli, namely the presence of predators or prey (Dutertre et al., 2014). Environmental variability could also help explain why some venom proteins, including latrotoxins, appeared to be encoded by transcripts with no venom gland expression, which we consider a prerequisite for presence in the venom. Although we controlled environmental conditions in the laboratory for spiders used in gene expression experiments in this study, it was not feasible to maintain spiders milked for venom under exactly the same conditions. The presence of proteins in venom in those spiders would likely indicate that the associated transcripts were expressed in the venom gland. The difference in venom gland expression between populations of spiders used for transcriptomics or proteomics could have an environmental, but also a genetic, origin. However, data from experiments assessing variability of expression in spider venom glands under different environmental conditions is as yet lacking, as is data on genetic structure among populations of *P. tepidariorum.*

In summary, through the generation and use of genomic, transcriptomic and proteomic data we find that venom complexity may be achieved through both gene duplication and complex patterns of alternative transcription. Patterns of transcript expression do not comport with simple predictions from a traditional model of venom evolution, and may reflect the interplay of environmental and genetic mechanisms. Though this study focused on a single spider species, we expect these findings will be broadly applicable across venomous taxa in explaining how diverse venom complements are generated.

## Supplementary Material

Data Sheet 1

STable1

STable2

STable3

STable4

STable5

STable6

Data Sheet 2

Data Sheet 3

Data Sheet 4

Data Sheet 5

Data Sheet 6

Data Sheet 7

Data Sheet 8

Data Sheet 9

## Figures and Tables

**TABLE 1 |:** Numbers of proteins corresponding to different known toxin or other category of interest in venom, or from a transcript upregulated in the venom gland, or both, as determined by BLAST homology.

Class	# In venom	# VGTup	# In venom and VGTup

Latrotoxin	33–38	51	21
Latrodectin	1–1	2	1
CRISP	1–2	3	1
ICK	1–1	3	1
Protease	3–7	25	4
Other enzyme*	3–7	17	4
LRR	6–9	11	1
Uncharacterized	24–34	144	19
Novel family	8–9	14	9

* lipase, amylase, hyaluronidase. Numbers separated by dashes indicate minimum and maximum estimates of the number of proteins of a given type, given ambiguity in the MS identifications for similar proteins. To calculate the number that are both in venom and are VGTup, we used the maximum estimate of distinct proteins in venom. CRISP, cysteine-rich secretory protein; ICK, inhibitory cysteine knot toxin; LRR, leucine-rich repeat protein; Novel Family, putative toxin family identified in Gendreau et al. (2017).

**TABLE 2 |:** Basic information for eight loci that contribute more than one protein to venom as assessed by tandem mass spectrometry.

Gene	BLAST ID	transcripts/proteins/ proteins in venom	Genomic coordinates

MSTRG.15150	Latrotoxin	4/4/2	Scf5bK3_2009:246682–283244
MSTRG.21390	Leucine-rich repeat	6/5/2	Scf5bK3_258:4313870–4351624
MSTRG.2390	None	7/5/2	Scf5bk3_112:476966–508466
MSTRG.25517	Uncharacterized	5/3/2	Scf5bK3_306:5509048–5555723
MSTRG.32970	Latrotoxin	8/6/2	Scf5bK3_440:829934–879481
MSTRG.35296	Chitinase	27/22/2	Scf5bK3_479:825520–993306
MSTRG.35640	Endothelin-converting enzyme	22/20/4	Scf5bK3_479:10697475–10953136
MSTRG.45528	Latrotoxin	23/17/7	Scf5bK3_756:633096–755920

Column 3 lists the total number of transcripts predicted at the locus, the number of distinct proteins predicted from these transcripts, and the total number identified in venom.
