# Supplementary material for: Alternative Transcription at Venom Genes and Its Role as a Complementary Mechanism for the Generation of Venom Complexity in the Common House Spider
Source: Front Ecol Evol. Author manuscript; Available in PMC 2019 Aug 20. (PMC6700725; doi:10.3389/fevo.2019.00085)
Supplement: Data Sheet 1 [file NIHMS1042230-supplement-Data_Sheet_1.PDF]

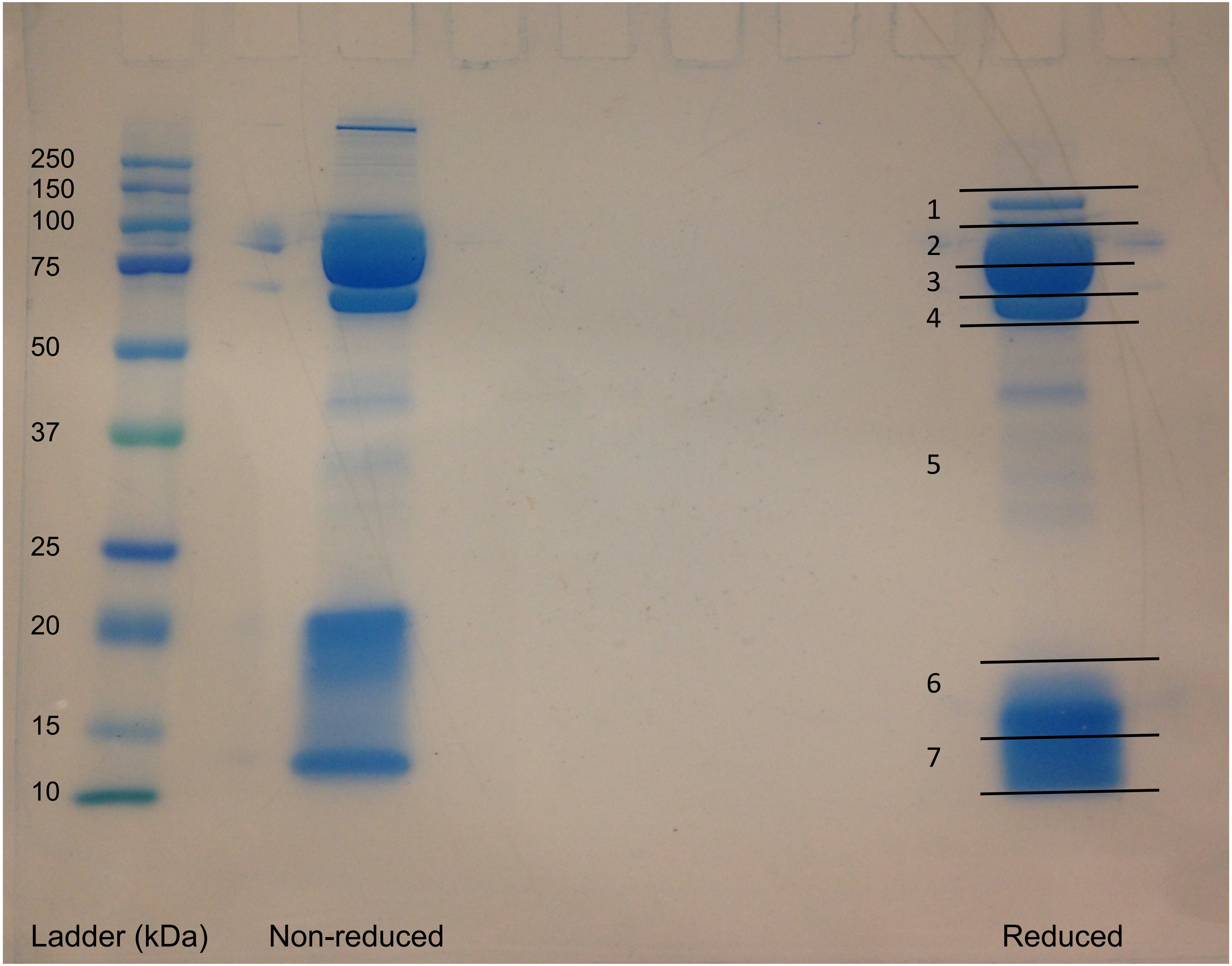

Figure S1. SDS PAGE gel loaded with 16 ug venom protein. Lines delineate numbered gel slices isolated for MS.
