# Supplementary material for: Alternative Transcription at Venom Genes and Its Role as a Complementary Mechanism for the Generation of Venom Complexity in the Common House Spider
Source: Front Ecol Evol. Author manuscript; Available in PMC 2019 Aug 20. (PMC6700725; doi:10.3389/fevo.2019.00085)
Supplement: STable4 [file NIHMS1042230-supplement-STable4.DOCX]

| **Library** | **Tissue** | **Source** | **Mapped reads** |
| --- | --- | --- | --- |
| Ven1 | Venom gland | This study | 35998058 / 18911545 |
| Ven2 | Venom gland | This study | 20250232 / 8607333 |
| Ven2139 | Venom gland | Gendreau et al. 2017 | 94010053 / 38765099 |
| Silk1 | Silk gland | This study | 77783575 / 27580446 |
| Silk2 | Silk gland | This study | 88839928 / 29824324 |
| Silk15A | Silk gland | Gendreau et al. 2017 | 95350914 / 43683575 |
| Ov3 | Ovary | This study | 27388186 / 5920755 |
| Ov4 | Ovary | This study | 30578785 / 6571939 |
| Ovi5k | Ovary | i5k project | 49580040 / 6214932 |
| Ceph1 | Cephalothorax | This study | 21425243 / 10628079 |
| Ceph2 | Cephalothorax | This study | 25607588 / 13520615 |

**Table S4**. Shown are library names within the study, the tissue from which the library was derived, the source of the data, and the number of reads that mapped to the dovetail assembly. The first value in the last column are reads that mapped as pairs, the second value is the number of unpaired reads mapped.
