# Supplementary material for: Alternative Transcription at Venom Genes and Its Role as a Complementary Mechanism for the Generation of Venom Complexity in the Common House Spider
Source: Front Ecol Evol. Author manuscript; Available in PMC 2019 Aug 20. (PMC6700725; doi:10.3389/fevo.2019.00085)
Supplement: Data Sheet 3 [file NIHMS1042230-supplement-Data_Sheet_3.PDF]

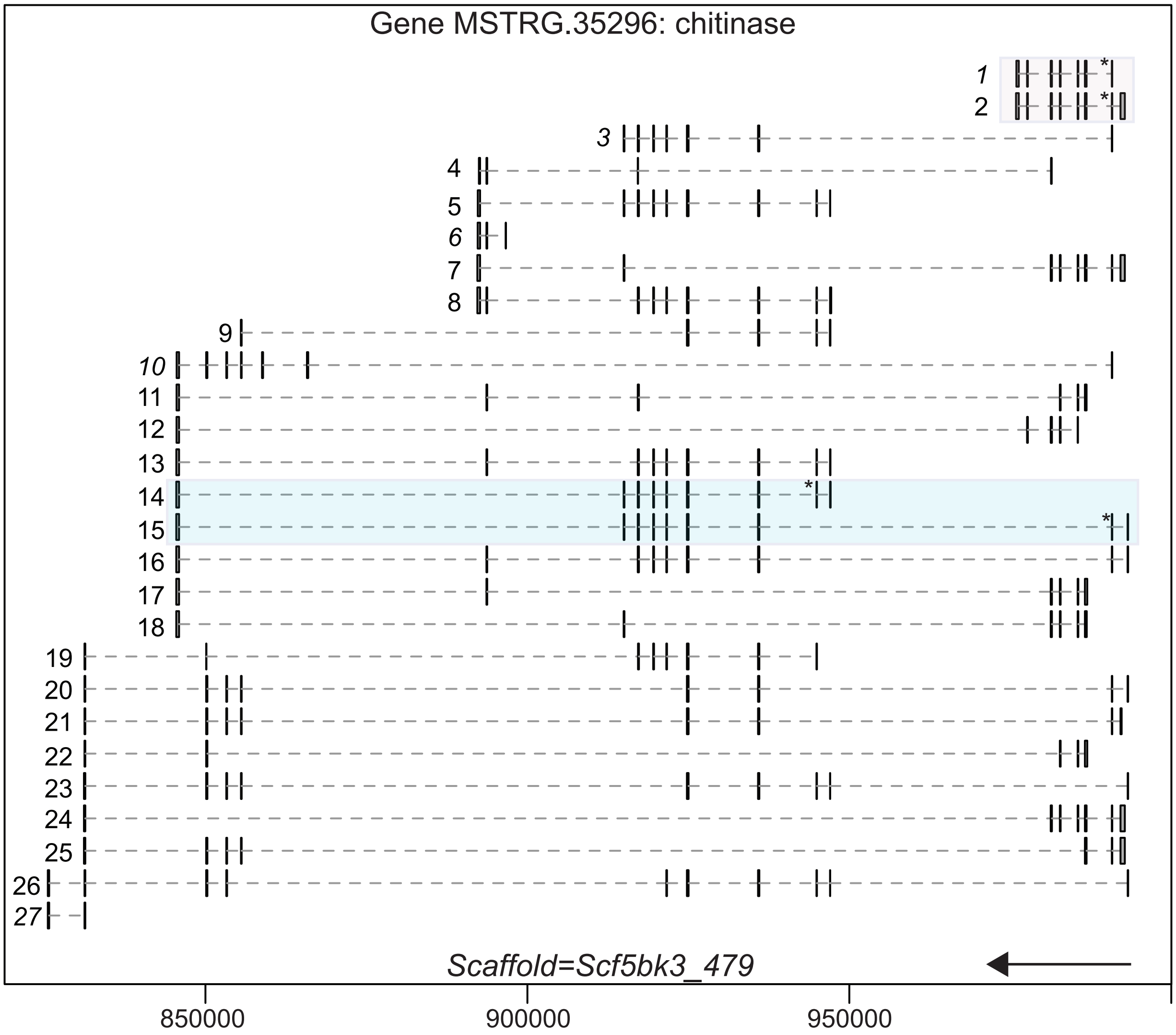

Transcript key: 1-aug3.g6418.t1, 2-MSTRG.35296.26, 3-aug3.g6417.t1, 4-MSTRG.35296.24, 5-MSTRG.35296.23, 6-aug3.g20179.t1 7-MSTRG.35296.21, 8-MSTRG.35296.20, 9-MSTRG.35296.19, 10-aug3.g20180.t1, 11-MSTRG.35296.17, 12-MSTRG.35296.16 13-MSTRG.35296.15, 14-MSTRG.35296.14, 15-MSTRG.35296.12, 16-MSTRG.35296.10, 17-MSTRG.35296.11, 18-MSTRG.35296.13 19-MSTRG.35296.9, 20-MSTRG.35296.6, 21-MSTRG.35296.8, 22-MSTRG.35296.7, 23-MSTRG.35296.3, 24-MSTRG.35296.5 25-MSTRG.35296.4, 26-MSTRG.35296.1, 27-aug3.g16938.t1

Figure S3. Above is the exon-intron structure of transcripts predicted for locus MSTRG.35296, with homology to chitinases. The two novel transcripts that produce a novel protein identified in venom by MS data are highlighted in light blue. A second set of two transcripts that each produce a second protein also identified in venom are shaded light purple. Transcripts are numbered in the figure due to space constraints and labels are below. Transcripts prefixed with MSTRG are novel to this study, whereas those prefixed with aug3 are from the original genome annotation, with numbers in italics. * indicates an exon producing the identical first 22 amino acids of predicted proteins from the two sets of two transcripts. The arrow indicates inferred direction of transcription. Spliced read counts for novel junctions are found in Table S3, but not included in the figure due to space considerations.
The alignment of the two distinct protein sequences identified in venom by Scaffold is shown below. Each protein sequence is encoded by two distinct transcripts. MSTRG.35296.12 and MSTRG.35296.14 are novel transcripts that encode the same novel protein by combining exons across genes in the original genome annotation, and this protein was identified in venom.

```
               **********************
MSTRG.35296.12 MDLKAISVILLLSVAFASAASKDRNNKKYRVVCYLGSWANYRPGAGKFLIEHIDPFLCTHVIYGFAKLAGNKIAAYDPYL
MSTRG.35296.14 MDLKAISVILLLSVAFASAASKDRNNKKYRVVCYLGSWANYRPGAGKFLIEHIDPFLCTHVIYGFAKLAGNKIAAYDPYL
MSTRG.35296.26 MDLKAISVILLLSVAFASAASK----------------------------------------------------------
aug3.g6418.t1  MDLKAISVILLLSVAFASAASK----------------------------------------------------------

                       **:**********************:******* ***:********:***************:*********
MSTRG.35296.12 DLKENWGLGAFQRFNALKKANPNLTTLIAIGGWNEGSKKYSEMAANPAARATFIQSVISFCEKYGFDGLDMDWEYPANRG
MSTRG.35296.14 DLKENWGLGAFQRFNALKKANPNLTTLIAIGGWNEGSKKYSEMAANPAARATFIQSVISFCEKYGFDGLDMDWEYPANRG
MSTRG.35296.26 --------GAYQRFNALKKANPNLTTLIAIGGWDEGSKKYSAMAADPAARATFIKSVISFCEKYGFDGLDVDWEYPANRG
aug3.g6418.t1  --------GAYQRFNALKKANPNLTTLIAIGGWDEGSKKYSAMAADPAARATFIKSVISFCEKYGFDGLDVDWEYPANRG

               *********..***********:***************:*******:**:**********:****::*************
MSTRG.35296.12 GKPEDKENFVALLRELKAAFASHGYLLTAAVSAGVKTMDTAYNIPEVSKYLDFINVMAYDLHGSWEKVVGHNAPMRVRPE
MSTRG.35296.14 GKPEDKENFVALLRELKAAFASHGYLLTAAVSAGVKTMDTAYNIPEVSKYLDFINVMAYDLHGSWEKVVGHNAPMRVRPE
MSTRG.35296.26 GKPEDKENFAVLLRELKAAFASNGYLLTAAVSAGVKTMETAYNIPEISKHLDFINVMAYDFHGSWDEVVGHNAPMRVRPE
aug3.g6418.t1  GKPEDKENFAVLLRELKAAFASNGYLLTAAVSAGVKTMETAYNIPEISKHLDFINVMAYDFHGSWDEVVGHNAPMRVRPE

               ***.:****************************** *********************.*****: ****:**********
MSTRG.35296.12 EKDDERTLNVEYAINYWINKGAPRNKIVLGMGLYGRSFTLTDPSKTDLGSPAKGPGRGGPITKEPGMLGYNEICLNLKQG
MSTRG.35296.14 EKDDERTLNVEYAINYWINKGAPRNKIVLGMGLYGRSFTLTDPSKTDLGSPAKGPGRGGPITKEPGMLGYNEICLNLKQG
MSTRG.35296.26 EKDGQRTLNVEYAINYWINKGAPRNKIVLGMGLYGTSFTLTDPSKTDLGSPAKGPGRAGPITKKDGMLGFNEICLNLKQG
aug3.g6418.t1  EKDGQRTLNVEYAINYWINKGAPRNKIVLGMGLYGTSFTLTDPSKTDLGSPAKGPGRAGPITKKDGMLGFNEICLNLKQG

               ****** *******************************************************:****::*********:*
MSTRG.35296.12 WKEVVPDKVDAPYAYSGDQWVGYDDKKSIGIKVDYLIKEGLGGGMVWSLETDDFRGNCYGEKYPLLAHIYTKLNGPVVRP
MSTRG.35296.14 WKEVVPDKVDAPYAYSGDQWVGYDDKKSIGIKVDYLIKEGLGGGMVWSLETDDFRGNCYGEKYPLLAHIYTKLNGPVVRP
MSTRG.35296.26 WKEVVPAKVDAPYAYSGDQWVGYDDKKSIGIKVDYLIKEGLGGGMVWSLETDDFRGNCYGEKFPLLSQIYTKLNGPVVKP
aug3.g6418.t1  WKEVVPAKVDAPYAYSGDQWVGYDDKKSIGIKVDYLIKEGLGGGMVWSLETDDFRGNCYGEKFPLLSQIYTKLNGPVVKP

               ******:******** .** ****.*************:******* *::******* ******: *******:****
MSTRG.35296.12 TINPNIRPTVGPKPTGKPKGFTCPGEGYFRDPHNCSVFHYCVQVSKWEMARHTYNCGADSAFDEASKTCVLRKHVPGC
MSTRG.35296.14 TINPNIRPTVGPKPTGKPKGFTCPGEGYFRDPHNCSVFHYCVQVSKWEMARHTYNCGADSAFDEASKTCVLRKHVPGC
MSTRG.35296.26 TINPNIKPTVGPKPTETPKEFTCPSEGYFRDPHNCSVFYYCVQVSKGELTRHTYNCGEDSAFDETLKTCVLRKNVPGC
aug3.g6418.t1  TINPNIKPTVGPKPTETPKEFTCPSEGYFRDPHNCSVFYYCVQVSKGELTRHTYNCGEDSAFDETLKTCVLRKNVPGC
```
