# Supplementary material for: Alternative Transcription at Venom Genes and Its Role as a Complementary Mechanism for the Generation of Venom Complexity in the Common House Spider
Source: Front Ecol Evol. Author manuscript; Available in PMC 2019 Aug 20. (PMC6700725; doi:10.3389/fevo.2019.00085)
Supplement: Data Sheet 5 [file NIHMS1042230-supplement-Data_Sheet_5.PDF]

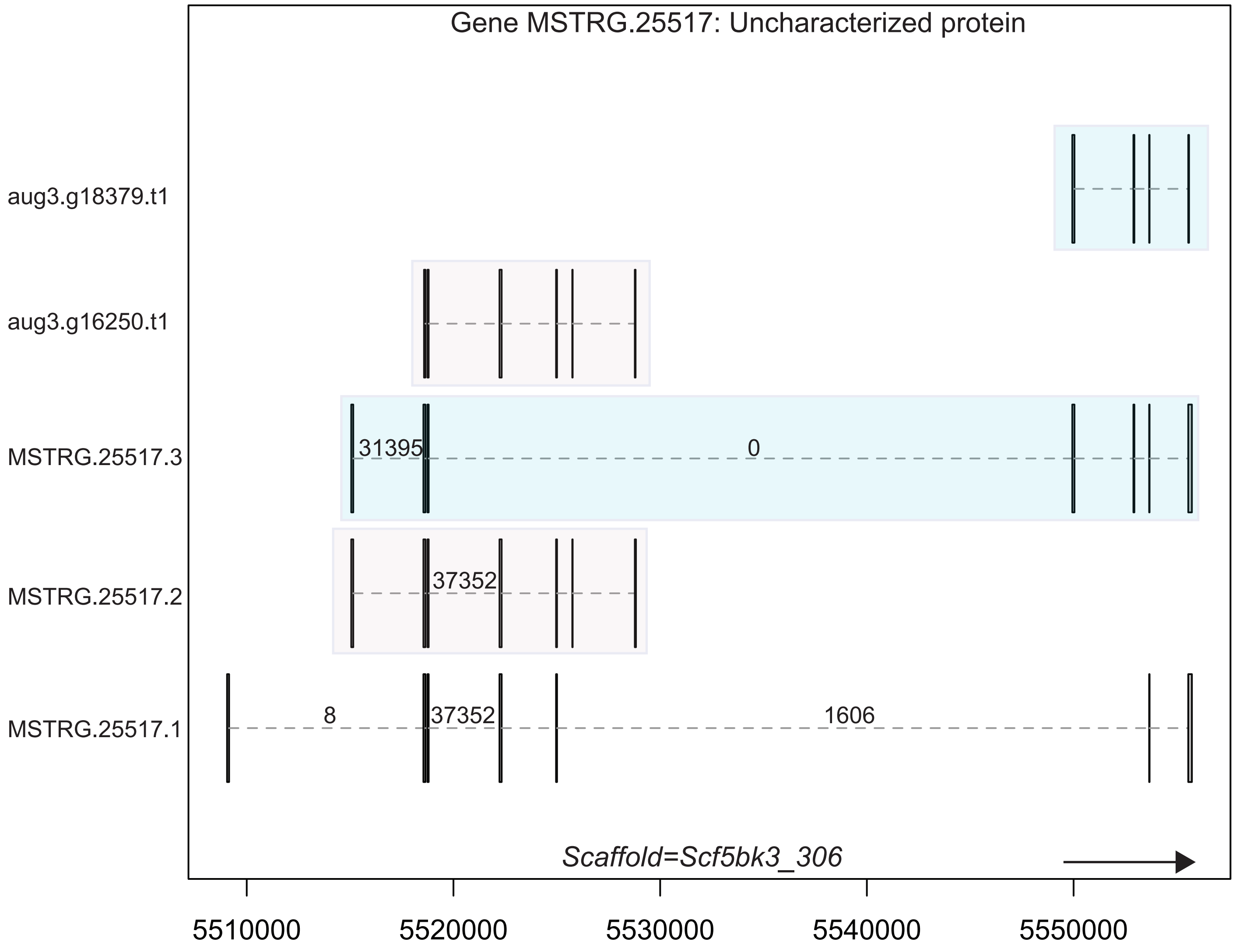


```
               ********************************************************************************
MSTRG.25517.2  MYAIWIILVFCALSVSAGPDVICPKVTEELGADFDKCDEKDPERVVKVIRACHKEVLPDKEPNKYNYFVAACKDPTLWKT
MSTRG.25517.1  MYAIWIILVFCALSVSAGPDVICPKVTEELGADFDKCDEKDPERVVKVIRACHKEVLPDKEPNKYNYFVAACKDPTLWKT
MSTRG.25517.3  MYAIWIILVFCALSVSAGPDVICPKVTEELGADFDKCDEKDPERVVKVIRACHKEVLPDKEPNKYNYFVAACKDPTLWKT

               **:*************************:*******
MSTRG.25517.2  FNKCFTRDLPVDQFPDSDFANSAACYEELNKKYDIK
MSTRG.25517.1  FNKCFTRDLPVDQFPDSDFANSAACYEEMNKKYDIK
MSTRG.25517.3  FNQCFTRDLPVDQFPDSDFANSAACYEEMNKKYDIK
```

Figure S5. The exon-intron structure of predicted transcripts at gene MSTRG.25517 (uncharacterized protein) is shown at top. The alignment of all distinct proteins predicted at this locus is shown at bottom. Transcripts producing identical proteins are indicated by shaded boxes of the same color. The arrow indicates inferred direction of transcription. Numbers over introns represent spliced read counts for novel junctions across all libraries, where space allows. Values for other novel introns are found in Table S3.

.
