# Supplementary material for: Alternative Transcription at Venom Genes and Its Role as a Complementary Mechanism for the Generation of Venom Complexity in the Common House Spider
Source: Front Ecol Evol. Author manuscript; Available in PMC 2019 Aug 20. (PMC6700725; doi:10.3389/fevo.2019.00085)
Supplement: Data Sheet 6 [file NIHMS1042230-supplement-Data_Sheet_6.PDF]

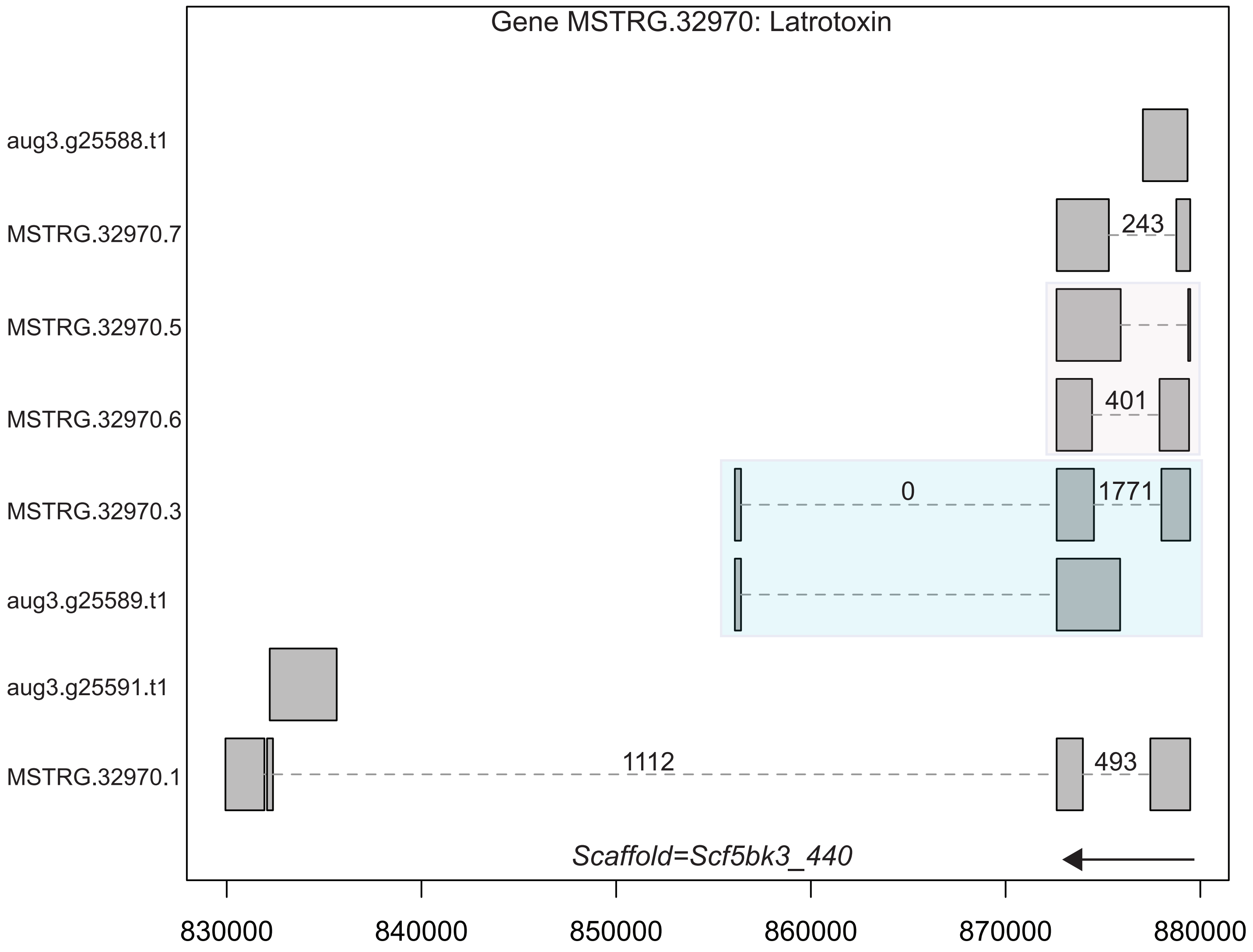

Figure S6. The exon-intron structure of predicted transcripts at gene MSTRG.32970 (latrotoxin) is shown above. The alignment of all distinct proteins predicted at this locus is shown below. Transcripts producing identical proteins are indicated by shaded boxes of the same color. The arrow indicates inferred direction of transcription. Numbers over introns represent spliced read counts for novel junctions across all libraries, where space allows. Values for other novel introns are found in Table S3.
.

```
                  *::.    :* ******    **::*::*:*:****: ** **.:*** ****** ***.*:**:**:*:**:** **::*
aug3.g25591.t1 MRIDTTSLASAVAKRDTDDDADVNADYEKSLEEIETRKKICKGFEYAEHTTGLVGNLIDDLPDLPLITDTVKVVLSGITA
MSTRG.32970.1  MHVSQMVVALAVAKRD----ADLDAEFEESMEEIESMKKKCKALEYATHTTGLVTNLIGDIPDIPLVTETVQVVASGVAA
MSTRG.32970.3  MHVSQMVVALAVAKRD----ADLDAEFEESMEEIESMKKKCKALEYATHTTGLVTNLIGDIPDIPLVTETVQVVASGVAA
MSTRG.32970.6  MHVSQMVVALAVAKRD----ADLDAEFEESMEEIESMKKKCKALEYATHTTGLVTNLIGDIPDIPLVTETVQVVASGVAA
MSTRG.32970.7  MHVSQMVVALAVAKRD----ADLDAEFEESMEEIESMKKKCKALEYATHTTGLVTNLIGDIPDIPLVTETVQVVASGVAA
aug3.g25588.t1 MHVSQMVVALAVAKRD----ADLDAEFEESMEEIESMKKKCKALEYATHTTGLVTNLIGDIPDIPLVTETVQVVASGVAA

               : *:**:**:**.***.*.  **:***.**:***.::*:*******.**:: *  * *  :***** **.****.**::*
aug3.g25591.t1 ILHIAEFGMDIAQTAMRCGAVNFDEVKVIMNKRFDDVDKKLDKMTEVMEKLDEAITITLRSVEKTRAEMNWGFKTILASL
MSTRG.32970.1  VAHVAEYGMNIASTAMECDDWNFEEVKAIMEKRFSEIDRKLDKMTEAMERVTEMATKTYDAVEKTREEMSWGFKNILTTL
MSTRG.32970.3  VAHVAEYGMNIASTAMECDDWNFEEVKAIMEKRFSEIDRKLDKMTEAMERVTEMATKTYDAVEKTREEMSWGFKNILTTL
MSTRG.32970.6  VAHVAEYGMNIASTAMECDDWNFEEVKAIMEKRFSEIDRKLDKMTEAMERVTEMATKTYDAVEKTREEMSWGFKNILTTL
MSTRG.32970.7  VAHVAEYGMNIASTAMECDDWNFEEVKAIMEKRFSEIDRKLDKMTEAMERVTEMATKTYDAVEKTREEMSWGFKNILTTL
aug3.g25588.t1 VAHVAEYGMNIASTAMECDDWNFEEVKAIMEKRFSEIDRKLDKMTEAMERVTEMATKTYDAVEKTREEMSWGFKNILTTL

               **:*: .**.::********* ::*:.***::** ***.::*.*****:  **************** *****.:*:*.
aug3.g25591.t1 KNHEIQTILSKINKFSRYFEEKKDEINKLPKDQFVFRLKEEDGILDYLQKIRKPEGLHSDLLELMDKNTNYAIPKNAEDN
MSTRG.32970.1  KNREVLPILKRMNKFSRYFEEMRNELSKLPENQFRFRLTKKDNILDYLKIARKPEGLHSDLLELMDKNYNYAIPTDAKDS
MSTRG.32970.3  KNREVLPILKRMNKFSRYFEEMRNELSKLPENQFRFRLTKKDNILDYLKIARKPEGLHSDLLELMDKNYNYAIPTDAKDS
MSTRG.32970.6  KNREVLPILKRMNKFSRYFEEMRNELSKLPENQFRFRLTKKDNILDYLKIARKPEGLHSDLLELMDKNYNYAIPTDAKDS
MSTRG.32970.7  KNREVLPILKRMNKFSRYFEEMRNELSKLPENQFRFRLTKKDNILDYLKIARKPEGLHSDLLELMDKNYNYAIPTDAKDS
aug3.g25588.t1 KNREVLPILKRMNKFSRYFEEMRNELSKLPENQFRFRLTKKDNILDYLKIARKPEGLHSDLLELMDKNYNYAIPTDAKDS

               *****************:*********:***:*:**:*:* .**::*:*::***:****** **:*:**:**:::*****
aug3.g25591.t1 KAFQALYALFYGTQTYASVMFFLLKQHSYLANYFYQEGKDADFNDEFDILIATFKEFKTSLIGSKGLINKVVQTLDEVKN
MSTRG.32970.1  KAFQALYALFYGTQTYAAVMFFLLKQHAYLADYYYQDGQDKKFNEQFEIMVATFNEFKTSLTGSHGVINEVVKSVDEVKN
MSTRG.32970.3  KAFQALYALFYGTQTYAAVMFFLLKQHAYLADYYYQDGQDKKFNEQFEIMVATFNEFKTSLTGSHGVINEVVKSVDEVKN
MSTRG.32970.6  KAFQALYALFYGTQTYAAVMFFLLKQHAYLADYYYQDGQDKKFNEQFEIMVATFNEFKTSLTGSHGVINEVVKSVDEVKN
MSTRG.32970.7  KAFQALYALFYGTQTYAAVMFFLLKQHAYLADYYYQDGQDKKFNEQFEIMVATFNEFKTSLTGSHGVINEVVKSVDEVKN
aug3.g25588.t1 KAFQALYALFYGTQTYAAVMFFLLKQHAYLADYYYQDGQDKKFNEQFEIMVATFNEFKTSLTGSHGVINEVVKSVDEVKN

               * **  *:::** ***:* . *.**:*:*:.* * :***.*:**:* ** **:***** **:**:::****::. :***
aug3.g25591.t1 KPFILFVEDQLYADISKRQGSLRELQSNIANMTLYVIEDVPDPVLDIDFDEPIIPSNYGDWDDKARVKYAVQLRK-NGTY
MSTRG.32970.1  KDFIKDVKNELYEDISRRGSILNELKSKITGMELNIIEDTPEPVFDYDFTEPVIPSNYLDWEDKSKIRYAVQFKSTDGTY
MSTRG.32970.3  KDFIKDVKNELYEDISRRGSILNELKSKITGMELNIIEDTPEPVFDYDFTEPVIPSNYLDWEDKSKIRYAVQFKSTDGTY
MSTRG.32970.6  KDFIKDVKNELYEDISRRGSILNELKSKITGMELNIIEDTPEPVFDYDFTEPVIPSNYLDWEDKSKIRYAVQFKSTDGTY
MSTRG.32970.7  KDFIKDVKNELYEDISRRGSILNELKSKITGMELNIIEDTPEPVFDYDFTEPVIPSNYLDWEDKSKIRYAVQFKSTDGTY
aug3.g25588.t1 KDFIKDVKNELYEDISRRGSILNELKSKITGMELNIIEDTPEPVFDYDFTEPVIPSNYLDWEDKSKIRYAVQFKSTDGTY

               *:**:* *.:******** :*::***  *.******:** **** *:*****:****:*****:*:::*:*
aug3.g25591.t1 SKFSDWTESVKVDGKANPFLKINRDEYGRDRLVFRKFNDEKPQLAGILTKSQMEFRDIDRDLYNAAKSKNQNISLEYIPK
MSTRG.32970.1  SRFSQWMEPIKVDGKANPTIKVHRDERERSRLVFRKINDGKPQLIGVLTKSQVEFRDVDRDLYEASRAKDQ---------
MSTRG.32970.3  SRFSQWMEPIKVDGKANPTIKVHRDERERSRLVFRKINDGKPQLIGVLTKSQVEFRDVDRDLYEASRAKDQ---------
MSTRG.32970.6  SRFSQWMEPIKVDGKANPTIKVHRDERERSRLVFRKINDGKPQLIGVLTKSQVEFRDVDRDLYEASRAKDQ---------
MSTRG.32970.7  SRFSQWMEPIKVDGKANPTIKVHRDERERSRLVFRKINDGKPQLIGVLTKSQVEFRDVDRDLYEASRAKDQ---------
aug3.g25588.t1 SRFSQWMEPIKVDGKANPTIKVHRDERERSRLVFRKINDGKPQLIGVLTKSQVEFRDVDRDLYEASRAKDQ---------

                                       ..*   *.   *::.    :                                :::*
aug3.g25591.t1 LIEEGAYIRQVFEMNRSVMHAAAESGNVKIAFHYLLDVPDNL----------------------------------LVNA
MSTRG.32970.1  -----------------------SKGLECIP--KLIEAGAYISARFEMNRCVMHGAAESGNAKIALRYLIDDRIGEILDA
MSTRG.32970.3  -----------------------SKGLECIP--KLIEAGAYISARFEMNRCVMHGAAESGNAKIALRYLIDDRIGEILDA
MSTRG.32970.6  -----------------------SKGLECIP--KLIEAGAYISARFEMNRCVMHGAAESGNAKIALRYLIDDRIGEILDA
MSTRG.32970.7  -----------------------SKGLECIP--KLIEAGAYISARFEMNRCVMHGAAESGNAKIALRYLIDDRIGEILDA
aug3.g25588.t1 -----------------------SKGLECIP--KLIEAGAYISARFEMNRCVMHGAAESGNAKIALRYLIDDRIGEILDA

                **:*:*******.******::**   :.****** *:* *****:**::*: *:.: **: :: :***************
aug3.g25591.t1 VDDKGYTPMHVAADTKNAGFINFLITHSADVNAQTTSEKLTPLHLAARKGYSIAVNYLLSSDKIEINKPEKSGYTPLHYA
MSTRG.32970.1  KDDNGFTPMHVAASTKNAGFVKFLGDRNADVNAQ-TAEGLTPLHIAAKNGFYISAQNLLAYERTQINKPEKSGYTPLHYA
MSTRG.32970.3  KDDNGFTPMHVAASTKNAGFVKFLGDRNADVNAQ-TAEGLTPLHIAAKNGFYISAQNLLAYERTQINKPEKSGYTPLHYA
MSTRG.32970.6  KDDNGFTPMHVAASTKNAGFVKFLGDRNADVNAQ-TAEGLTPLHIAAKNGFYISAQNLLAYERTQINKPEKSGYTPLHYA
MSTRG.32970.7  KDDNGFTPMHVAASTKNAGFVKFLGDRNADVNAQ-TAEGLTPLHIAAKNGFYISAQNLLAYERTQINKPEKSGYTPLHYA
aug3.g25588.t1 KDDNGFTPMHVAASTKNAGFVKFLGDRNADVNAQ-TAEGLTPLHIAAKNGFYISAQNLLAYERTQINKPEKSGYTPLHYA

               ****.***: ***.::*.*** *******************************:*:::.*********:*** :******
aug3.g25591.t1 VRGTPKTIQTLLAKDQISVNARSDFGLTPFHLAVMKGNREICDALLSSGKVEVNAGDKNNMTPLHFAAMEGNINMIQYLL
MSTRG.32970.1  VRGTAKTIRLLLADERIEVNALSDFGLTPFHLAVMKGNREICDALLSSGKVEVNTGNRDGMTPLHFAALEGNADMIQYLL
MSTRG.32970.3  VRGTAKTIRLLLADERIEVNALSDFGLTPFHLAVMKGNREICDALLSSGKVEVNTGNRDGMTPLHFAALEGNADMIQYLL
MSTRG.32970.6  VRGTAKTIRLLLADERIEVNALSDFGLTPFHLAVMKGNREICDALLSSGKVEVNTGNRDGMTPLHFAALEGNADMIQYLL
MSTRG.32970.7  VRGTAKTIRLLLADERIEVNALSDFGLTPFHLAVMKGNREICDALLSSGKVEVNTGNRDGMTPLHFAALEGNADMIQYLL
aug3.g25588.t1 VRGTAKTIRLLLADERIEVNALSDFGLTPFHLAVMKGNREICDALLSSGKVEVNTGNRDGMTPLHFAALEGNADMIQYLL
```

```
                  ******* *: **.*. :****** ****:*:********                    : *.       :***:   :
aug3.g25591.t1    SGKKEVEGVEVNAVTSDTNWTPLYAAIYFQQEDAALELLKNEKVDISLTSKEKNTPLQLSI-ANGQMKVFEELLKKT-SD
MSTRG.32970.1     SGKKEVERVNPNAATPASNWTPLYFAIYFKQKDAALELLKSNNVDISISSKENVSPLQLSI-ANGLTEVFDELLKKN-PN
MSTRG.32970.3     SGKKEVERVNPNAATPASNWTPLYFAIYFKQKDAALELLKSNNVDISISSKENVSPLQLSI-ANGLTEVFDELLKKN-PN
MSTRG.32970.6     SGKKEVERVNPNAATPASNWTPLYFAIYFKQKDAALELLKSNNVDISISSKENVSPLQLSI-ANGLTEVFDELLKKN-PN
MSTRG.32970.7     SGKKEVERVNPNAATPASNWTPLYFAIYFKQKDAALELLKSNNVDISISSKENVSPLQLSI-ANGLTEVFDELLKKN-PN
aug3.g25588.t1    SGKKEVEGVNPNAVTPASNWTPLYFAIYFKQKDAALELLKXXXXXXXXXXXXXXXXXAYYVHARELNPDRLDLLKRDRKE

                  *  *
aug3.g25591.t1    LGSTTEKGLTALHVAALRPEADFIAKLLDRNANIDAKALDGNTPLHLAAKSDKMDPIILLINKGASLKHFNNKNLLPPYY
MSTRG.32970.1     LEATSNEGLTALHVAALRPETVFTSKLLDKNVNINSVSKDGSTPLHLASKADKKDQISFLISKGANMKLYDNQNFLPIQY
MSTRG.32970.3     LEATSNEGLTALHVAALRPETVFTSKLLDKNVNINSVSKDGSTPLHLASKADKKDQISFLISKGANMKLYDNQNFLPIQY
MSTRG.32970.6     LEATSNEGLTALHVAALRPETVFTSKLLDKNVNINSVSKDGSTPLHLASKADKKDQISFLISKGANMKLYDNQNFLPIQY
MSTRG.32970.7     LEATSNEGLTALHVAALRPETVFTSKLLDKNVNINSVSKDGSTPLHLASKADKKDQISFLISKGANMKLYDNQNFLPIQY
aug3.g25588.t1    LWLT----------------------------------------------------------------------------

aug3.g25591.t1    FVKNMNYEAMNAMVKQDPNILHFNTTEGIAVGQYCLDFVFFRIFDFYKNKDSFEYRKYKNLPPLEAFHKLGYYNLLRTL-
MSTRG.32970.1     SIKNINLQAMREAISQDPSIVDGHTQEGRDVWMYCLQFIIGAVSHYRRNQDSNEYRKYKDLEPFEVYNKLGYYDLLRIVV
MSTRG.32970.3     SIKNINLQAMREAISQDPSIVDGHTQEGRDVWMYCLQFIIGAVSHYRRNQDSNEYRKYKDLEPFEVYNKLGYYDLLRIVV
MSTRG.32970.6     SIKNINLQAMREAISQDPSIVDGHTQEGRDVWMYCLQFIIGAVSHYRRNQDSNEYRKYKDLEPFEVYNKLGYYDLLRIVV
MSTRG.32970.7     SIKNINLQAMREAISQDPSIVDGHTQEGRDVWMYCLQFIIGAVSHYRRNQDSNEYRKYKDLEPFEVYNKLGYYDLLRIVV
aug3.g25588.t1    --------------------------------------------------------------------------------

aug3.g25591.t1    --SYDEKLINGTIEMANDAVIMCLEIEKNPDKKQCSFTFPNFHQKRDINNVYSKLQLAQSSTGPVQNVMSPNLLGSKLAD
MSTRG.32970.1     NNSSTEAYVQATMEIADRMAGVCIEKEKYPNAKPCLLL--DVRSKRNIEAVDTKAYL-YPKIRPIKETIH-NLLETKLSN
MSTRG.32970.3     NNSSTEAYVQATMEIADRMAGVCIEKEKYPNAKPCLLL--DVRSKRNIEAVDTKAYL-YPKIRPIKETIH-NLLETKLSN
MSTRG.32970.6     NNSSTEAYVQATMEIADRMAGVCIEKEKYPNAKPCLLL--DVRSKRNIEAVDTKAYL-YPKIRPIKETIH-NLLETKLSN
MSTRG.32970.7     NNSSTEAYVQATMEIADRMAGVCIEKEKYPNAKPCLLL--DVRSKRNIEAVDTKAYL-YPKIRPIKETIH-NLLETKLSN
aug3.g25588.t1    --------------------------------------------------------------------------------

aug3.g25591.t1    PRKSFSNAKTSMVQNVDTNGMLLWLDLIVRKVTNEKYNMKLNAPMSALESQAEALKIVEKVSEFIDSVSDVPAEELIDLA
MSTRG.32970.1     SSSSFPNAKSSLVQSMDANGILLLVDLLVRKFTNEKYNMKLKAPMSALESQVTALEIVDKVSNFVDSVSDVKAEELIDLA
MSTRG.32970.3     SSSSFPNAKSSLVQSMDANGILLLVDLLVRKFTNEKYNMKLKAPMSALESQVTALEIVDKVSNFVDSVSDVKAEELIDLA
MSTRG.32970.6     SSSSFPNAKSSLVQSMDANGILLLVDLLVRKFTNEKYNMKLKAPMSALESQVTALEIVDKVSNFVDSVSDVKAEELIDLA
MSTRG.32970.7     SSSSFPNAKSSLVQSMDANGILLLVDLLVRKFTNEKYNMKLKAPMSALESQVTALEIVDKVSNFVDSVSDVKAEELIDLA
aug3.g25588.t1    --------------------------------------------------------------------------------

aug3.g25591.t1    KLHSDVYKSIEGGKSDIILSLLCDQLRSIVDPESMEDFFSALISDASQNQDFINNVKQRCLSEANKE-------------
MSTRG.32970.1     KLHSDVYKSIEGGKSDIILSLLCDQLRSIVDPESMEDFFSALISDASQNQDFINNVKQRCLSEANKE-------------
MSTRG.32970.3     KLHSDVYKSIEGHLQ----KFSDDFKLPFVDLENISIPNWIIVPFPAQNENVDINLQNELAELLSGLEAKTLFKNLTISK
MSTRG.32970.6     KLHSDVYKSIEGGN------------------------------------------------------------------
MSTRG.32970.7     KLHSDVYKSI----------------------------------------------------------------------
aug3.g25588.t1    --------------------------------------------------------------------------------

aug3.g25591.t1    -----------------------------------------
MSTRG.32970.1     -----------------------------------------
MSTRG.32970.3     FWTKMNIMQKYVKLYEIAQPSSYMVEVGFSHVNPILSTEIS
MSTRG.32970.6     -----------------------------------------
MSTRG.32970.7     -----------------------------------------
aug3.g25588.t1    -----------------------------------------
```
