# Supplementary material for: Alternative Transcription at Venom Genes and Its Role as a Complementary Mechanism for the Generation of Venom Complexity in the Common House Spider
Source: Front Ecol Evol. Author manuscript; Available in PMC 2019 Aug 20. (PMC6700725; doi:10.3389/fevo.2019.00085)
Supplement: Data Sheet 7 [file NIHMS1042230-supplement-Data_Sheet_7.PDF]

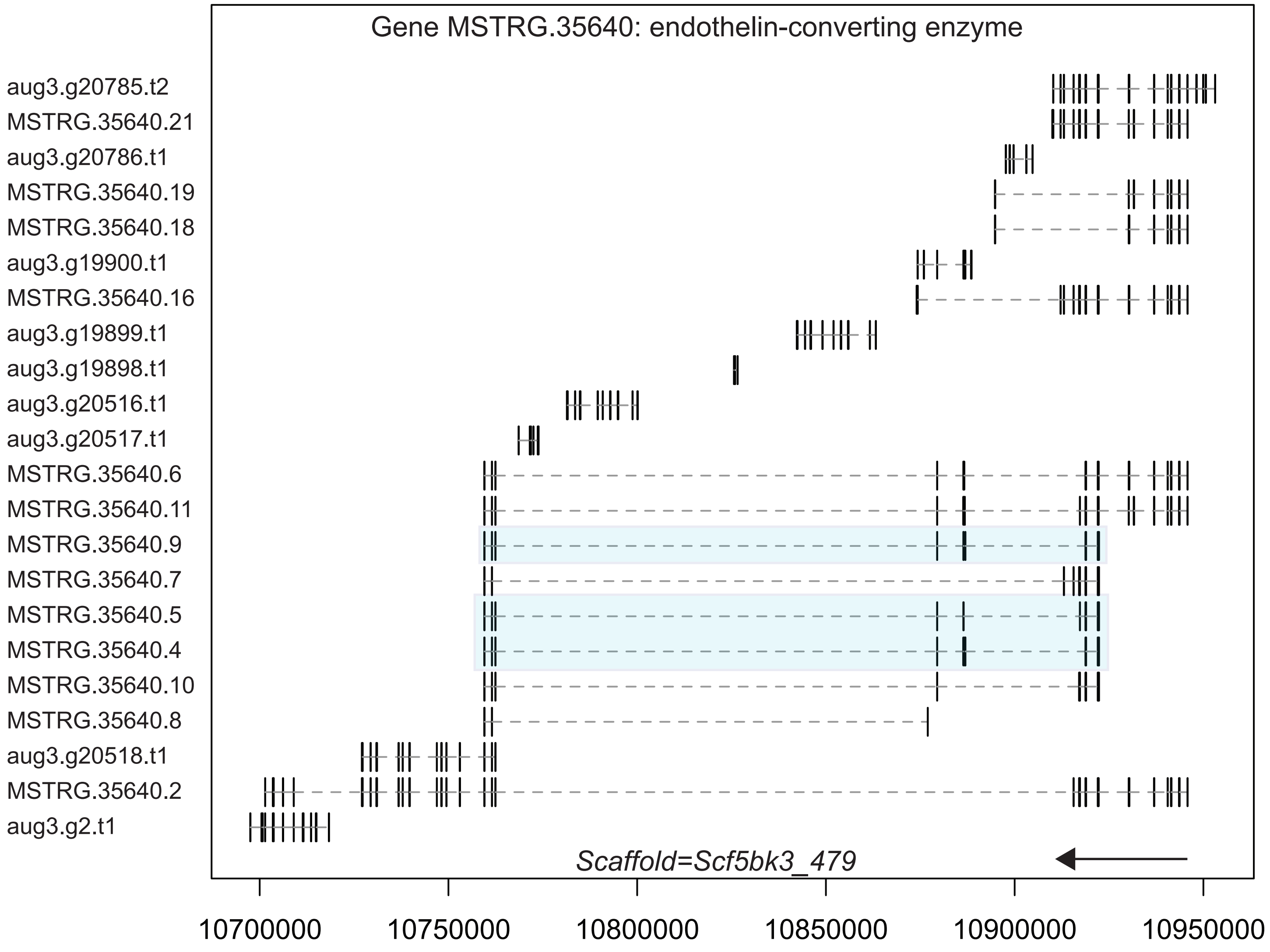

Figure S7. The exon-intron structure of predicted transcripts at gene MSTRG.35640 (endothelin-converting enzyme) is shown above. The alignment of all distinct proteins predicted at this locus is shown below. Transcripts producing identical proteins are indicated by shaded boxes of the same color. The arrow indicates inferred direction of transcription. Spliced read counts for novel junctions are found in Table S3, but not included in the figure due to space considerations.
.

```
aug3.g20785.t2 ----------------------------MFSISSRKHFYSSKFDMNPHWWNRRTRLERILCFVTITTLLILLMMAATFAV
MSTRG.35640.18 --------------------------------------------------------------------------------
MSTRG.35640.16 --------------------------------------------------------------------------------
MSTRG.35640.21 --------------------------------------------------------------------------------
aug3.g26606.t1 --------------------------------------------------------------------------------
MSTRG.35640.2  --------------------------------------------------------------------------------
aug3.g20518.t1 MILSDPHSPGEFRVNGPMSNLKEFSAAFGCATETKMNPDHKCQVWNSICGNHHMKFERTLYFFIVTSLLMTLTIPAVFG-
MSTRG.35640.4  --------------------------------------------------------------------------------
MSTRG.35640.6  --------------------------------------------------------------------------------
MSTRG.35640.7  --------------------------------------------------------------------------------
MSTRG.35640.8  --------------------------------------------------------------------------------
MSTRG.35640.10 --------------------------------------------------------------------------------
MSTRG.35640.11 --------------------------------------------------------------------------------
aug3.g20517.t1 --------------------------------------------------------------------------------
aug3.g20516.t1 --------------------------------------------------------------------------------
aug3.g19898.t1 --------------------------------------------------------------------------------
aug3.g19899.t1 --------------------------------------------------------------------------------
aug3.g19900.t1 --------------------------------------------------------------------------------
MSTRG.35640.19 --------------------------------------------------------------------------------
aug3.g20786.t1 ---------------------------MFSSSPREQFHSSKFIGMSSICGNRHMKLEKTLYFFLVASLLMTLTIPAVFG-

aug3.g20785.t2 FGYSYQTDAEKSAFVPSESRRFARQVNAEEVCNTPGCIETAEKIKQNLDESVDPCDNFYKFACGNWIKTHPIREDR----
MSTRG.35640.18 --------------------------------------------------------------------------------
MSTRG.35640.16 --------------------------------------------------------------------------------
MSTRG.35640.21 --------------------------------------------------------------------------------
aug3.g26606.t1 --------------------------------------------------------------------------------
MSTRG.35640.2  --------------------------------------------------------------------------------
aug3.g20518.t1 HNYRKKIDAIQTRFVPLNSPRFVQEANEEAICTTPGCVRTAEEIIQILDEKVNPCDNFYKFACGGWINKHSIPDDKAMMS
MSTRG.35640.4  --------------------------------------------------------------------------------
MSTRG.35640.6  --------------------------------------------------------------------------------
MSTRG.35640.7  --------------------------------------------------------------------------------
MSTRG.35640.8  --------------------------------------------------------------------------------
MSTRG.35640.10 --------------------------------------------------------------------------------
MSTRG.35640.11 --------------------------------------------------------------------------------
aug3.g20517.t1 --------------------------------------------------------------------------------
aug3.g20516.t1 -----------------------------------------------------------------------------MMS
aug3.g19898.t1 --------------------------------------------------------------------------------
aug3.g19899.t1 -----------------------------------------------------------------------------MLS
aug3.g19900.t1 --------------------------------------------------------------------------------
MSTRG.35640.19 --------------------------------------------------------------------------------
aug3.g20786.t1 HNYRKKIDTIQTRFVPLNSPRFEQEANEEAICTTPGCVRTAEEIIQILDEKVNPCDNFYKFACGGWINKHSIPDDKAMIS

aug3.g20785.t2 ---------------PESSVFYDVDDELSAQLKDFYDSCMNLDGIENSGSQPLQTVLTTLGGWPAVVGDSWDGSTFDWME
MSTRG.35640.18 ---------------------------------------MNLDGIENSGSQPLQTVLTTLGGWPAVVGDSWDGSTFDWME
MSTRG.35640.16 ---------------------------------------MNLDGIENSGSQPLQTVLTTLGGWPAVVGDSWDGSTFDWME
MSTRG.35640.21 ---------------------------------------MNLDGIENSGSQPLQTVLTTLGGWPAVVGDSWDGSTFDWME
aug3.g26606.t1 --------------------------------------------------------------------------------
MSTRG.35640.2  ---------------------------------------MNLDGIENSGSQPLQTVLTTLGGWPAVVGDSWDGSTFDWME
aug3.g20518.t1 VFHQVRDVLNLQLKGLLQKPSDESEPKSIKM-------------------------------------------------
MSTRG.35640.4  --------------------------------------------------------------------------------
MSTRG.35640.6  ---------------------------------------MNLDGIENSGSQPLQTVLTTLGGWPAVVGDSWDGSTFDWME
MSTRG.35640.7  --------------------------------------------------------------------------------
MSTRG.35640.8  --------------------------------------------------------------------------------
MSTRG.35640.10 --------------------------------------------------------------------------------
MSTRG.35640.11 ---------------------------------------MNLDGIENSGSQPLQTVLTTLGGWPAVVGDSWDGSTFDWME
aug3.g20517.t1 --------------------------------------------------------------------------------
aug3.g20516.t1 VFHQVRDVLNLQLKGLLQKPSDESEPKSIKMVKDMYNSCLDLDILEKSGSKPLQEVLQKLGGWPVVEGDKWDGSNFDWMD
aug3.g19898.t1 --------------------------------------------------------------------------------
aug3.g19899.t1 VFQQVEDVLNLQLKGLLQKPSDESEPKSIKMVKDMYNSCLDLDILEKSGSKPLQEVLQKLGGWPVVEGDKWDGSNFDWMD
aug3.g19900.t1 --------------------------------------------------------------------------------
MSTRG.35640.19 ---------------------------------------MNLDGIENSGSQPLQTVLTTLGGWPAVVGDSWDGSTFDWME
aug3.g20786.t1 VFQQVQDILNLQLKGLLQKPSDESEPKSIKMVKDMYNSCLDLGTFIRIILQIK---------------------------
```

```
aug3.g20785.t2  TLFKMRNLGYDHSILITTSVSTDFKNSTVHTIEMDQTSLGMPNREYYMKGLNDSATQAYFNLMVKAAKKLGANEQTVENE
MSTRG.35640.18  TLFKMRNLGYDHSILITTSVSTDFKNSTVHTIEMDQTSLGMPNREYYMKGLNDSATQAYFNLMVKAAKKLGANEQTVENE
MSTRG.35640.16  TLFKMRNLGYDHSILITTSVSTDFKNSTVHTIEMDQTSLGMPNREYYMKGLNDSATQAYFNLMVKAAKKLGANEQTVENE
MSTRG.35640.21  TLFKMRNLGYDHSILITTSVSTDFKNSTVHTIEMDQTSLGMPNREYYMKGLNDSATQAYFNLMVKAAKKLGANEQTVENE
aug3.g26606.t1  --------------------------------------------------------------------------------
MSTRG.35640.2   TLFKMRNLGYDHSILITTSVSTDFKNSTVHTIEMDQTSLGMPNREYYMKGLNDSATQAYFNLMVKAAKKLGANEQTVENE
aug3.g20518.t1  ---------------------------------LDQTSLGMPDRTYLVSGLSDSGTKAYFNLMVKAAKKLGANEETVEKE
MSTRG.35640.4   --------------------------------------------------------------------------------
MSTRG.35640.6   TLFKMRNLGYDHSILITTSVSTDFKNSTVHTIEMDQTSLGMPNREYYMKGLNDSATQAYFNLMVKAAKKLGANEQTVENE
MSTRG.35640.7   --------------------------------------------------------------------------------
MSTRG.35640.8   --------------------------------------------------------------------------------
MSTRG.35640.10  --------------------------------------------------------------------------------
MSTRG.35640.11  TLFKMRNLGYDHSILITTSVSTDFKNSTVHTIEMDQTSLGMPNREYYMKGLNDSATQAYFNLMVKAAKKLGANEQTVENE
aug3.g20517.t1  --------------------------------------------------------------------------------
aug3.g20516.t1  TLFAFRKHGYDFSILIELSVTIDLKNNAVHTIYLDQTSLGMPDRTYLVNGLSDSGTKAYYNLMVKAAKKLGANEETVEKE
aug3.g19898.t1  --------------------------------------------------------------------------------
aug3.g19899.t1  TLFAFRKHGYDFSILIELSVTIDLKNNAVHTIYLDQTSLGMPDRTYLVNGLSDSGTKAYFNLMVKAAKKLGANEETVEKE
aug3.g19900.t1  --------------------------------------------------------------------------------
MSTRG.35640.19  TLFKMRNLGYDHSILITTSVSTDFKNSTVHTIEMDQTSLGMPNREYYMKGLNDSATQAYFNLMVKAAKKLGANEQTVENE
aug3.g20786.t1  --------------------------------------------------------------------------------

aug3.g20785.t2  LLQALNFEITLANNSLPDEERRDFDKMYHKYTIPQLRELVPQIDWIKYFNGLVADPITETETVLVDVPDFLKKFGELITT
MSTRG.35640.18  LLQALNFEITLANNSLPDEERRDFDKMYHKYTIPQLRELVPQIDWIKYFNGLVADPITETETVLVDVPDFLKKFGELITT
MSTRG.35640.16  LLQALNFEITLANNSLPDEERRDFDKMYHKYTIPQLRELVPQIDWIKYFNGLVADPITETETVLVDVPDFLKKFGELITT
MSTRG.35640.21  LLQALNFEITLANNSLPDEERRDFDKMYHKYTIPQLRELVPQIDWIKYFNGLVADPITETETVLVDVPDFLKKFGELITT
aug3.g26606.t1  --------------------------------------------------------------------------------
MSTRG.35640.2   LLQALNFEITLANNSLPDEERRDFDKMYHKYTIPQLRELVPQIDWIKYFNGLVADPITETETVLVDVPDFLKKFGELITT
aug3.g20518.t1  LLEALNFEITLANYSLPREERRDYDSMYHKYTISQLRELVPQIDWLKYLNGLLNDPITETETLIVMVANFLKQFGELIAK
MSTRG.35640.4   --------------------------------------------------------------------------------
MSTRG.35640.6   LLQALNFEITLANNSLPDEERRDFDKMYHKYTIPQLRELVPQIDWIKYFNGLVADPITETETVLVDVPDFLKKFGELITT
MSTRG.35640.7   --------------------------------------------------------------------------------
MSTRG.35640.8   --------------------------------------------------------------------------------
MSTRG.35640.10  --------------------------------------------------------------------------------
MSTRG.35640.11  LLQALNFEITLANNSLPDEERRDFDKMYHKYTIPQLRELVPQIDWIKYFNGLVADPITETETVLVDVPDFLKKFGELITT
aug3.g20517.t1  --------------------------------------------------------------------------------
aug3.g20516.t1  LLEALNFEITLANYSLPREERRDYDSMYHKYTISQLRELVPQIDWLKYLNGLLNDPITETETLIVMVPNFLKQFGELIAK
aug3.g19898.t1  --------------------------------------------------------------------------------
aug3.g19899.t1  LLEALNFEITLANYSLPREERRDYDTMYHKYTISQLRELVPQIDWLKYLNGLLNDPITDTETLIVMVPNFLKQFGELIAK
aug3.g19900.t1  --------------------------------------------------------------------------------
MSTRG.35640.19  LLQALNFEITLANNSLPDEERRDFDKMYHKYTIPQLRELVPQIDWIKYFNGLVADPITETETVLVDVPDFLKKFGELITT
aug3.g20786.t1  --------------------------------------------------------------------------------

aug3.g20785.t2  TDKRVVANYMMWRVVGQSLGSLSKDWRALKHEYSSALSGESAEAPRWETCLRSVNGHLGIAVSSYYIKHYFKGDSKQKV-
MSTRG.35640.18  TDKRVVANYMMWRVVGQSLGSLSKDWRALKHEYSSALSGESAEAPRWETCLRSVNGHLGIAVSSYYIKHYFKGDSKQKA-
MSTRG.35640.16  TDKRVVANYMMWRVVGQSLGSLSKDWRALKHEYSSALSGESAEAPRWETCLRSVNGHLGIAVSSYYIKHYFKGDSKQKV-
MSTRG.35640.21  TDKRVVANYMMWRVVGESLGSLSKDWRALKHEYSSALSGESAEKPRWETCLRSVNGHLGIAVSSYYIKHYFKGDSKQKV-
aug3.g26606.t1  ---------MMWRVVADSAGMLSKDWKALVQEYVLAITGEREEKPRWEQCVNSLRENLEIALSSYYIKHYFKGDSKQKAS
MSTRG.35640.2   TDKRVVANYMMWRVVGQSLGSLSKDWRALKHEYSSALSGESAEAPRWETCLRSVNGHLGIAVSSYYIKHYFKGDSKQKV-
aug3.g20518.t1  TDKRIVANYMMWRVVADSAGMLSKDWKALVQEYVLAITGEREEKPRWEQCVNSLRENLEIALSSYYIKHYFKGDSKQKAS
MSTRG.35640.4   --------------------------------------------------------------------------------
MSTRG.35640.6   TDKRVVANYMMWRVVGQSLGSLSKDWRALKHEYSSALSGESAEAPRWETCLRSVNGHLGIAVSSYYIKHYFKGDSKQKV-
MSTRG.35640.7   --------------------------------------------------------------------------------
MSTRG.35640.8   --------------------------------------------------------------------------------
MSTRG.35640.10  --------------------------------------------------------------------------------
MSTRG.35640.11  TDKRVVANYMMWRVVGESLGSLSKDWRALKHEYSSALSGESAEKPRWETCLRSVNGHLGIAVSSYYIKHYFKGDSKQKV-
aug3.g20517.t1  --------------------------------------------------------------------------------
aug3.g20516.t1  TDKRIVANYMMWRVVADSAGMLSKDWKALVQEYVLAITGEREEKPRWEQCVNSLSENLEIALSSYYIKHYFKGDSKQKAS
aug3.g19898.t1  --------------------------------------------------------------------------------
aug3.g19899.t1  TDKRIVANYMMWRVVADSAGMLSKDWKALVQEYVLAITGEREEKPRWEQCVNSLSENLEIALSSYYIKHYFKGDSKQKAS
aug3.g19900.t1  --------------------------------------------------------------------------------
MSTRG.35640.19  TDKRVVANYMMWRVVGESLGSLSKDWRALKHEYSSALSGESAEKPRWETCLRSVNGHLGIAVSSYYIKHYFKGDSKQKA-
aug3.g20786.t1  --------------------------------------------------------------------------------
```

```
aug3.g20785.t2 --------------------------------NSITPYIGYPQELLNESIVSDYYKALTVANENYFSNVLNIKKWKVDEE
MSTRG.35640.18 --------------------------------LEMVGYL-----------------------SKEFLNILNDIDW-MDSE
MSTRG.35640.16 --------------------------------NSITPYIGYPQELLNESIVSDYYKALTVANENYFSNVLNIKKWKVDEE
MSTRG.35640.21 --------------------------------NSITPYIGYPQELLNESIVSDYYKALTVANENYFSNVLNIKKWKVDEE
aug3.g26606.t1 EMVKYISNAFLEILKQIDWMDEETRRQAIEKANAIASYVGYPQELLDDSMVSQLYANLTVTNENYFMNNLNIRKWTTNYY
MSTRG.35640.2  --------------------------------NSITPYIGYPQELLNESIVSDYYKALTVANENYFSNVLNIKKWKVDEE
aug3.g20518.t1 EMVKYISNAFLEILKQIDWMDEETRRQAIEKANAIVSYVGYPQELLDDSLVSQLYENLTITNENYFMNNLNIRKWTTNYY
MSTRG.35640.4  --------------------------------------------------------------------------------
MSTRG.35640.6  --------------------------------NSITPYIGYPQELLNESIVSDYYKALTVANENYFSNVLNIKKWKVDEE
MSTRG.35640.7  --------------------------------------------------------------------------------
MSTRG.35640.8  --------------------------------------------------------------------------------
MSTRG.35640.10 --------------------------------------------------------------------------------
MSTRG.35640.11 --------------------------------NSITPYIGYPQELLNESIVSDYYKALTVANENYFSNVLNIKKWKVDEE
aug3.g20517.t1 --------------------------------------------------------------------------------
aug3.g20516.t1 EMVKYISNAFLEILKQIDWMDEETRRQAIEKANAIASYVGYPQELLDDSLVSQLYENLTITSENYFMNNLNIRKWTTNYY
aug3.g19898.t1 --------------------------------------------------------------------------------
aug3.g19899.t1 EMVKYISNAFLEILKQIDWMDEETRRQAIEKANAIASYVGYPQELLDDSLVSQLYENLTITNENYFTNNLNIRKWTTNYY
aug3.g19900.t1 --------------------------------------------------------------------------------
MSTRG.35640.19 --------------------------------LEMVGYL-----------------------SKEFLNILNDIDW-MDSE
aug3.g20786.t1 --------------------------------------------------------------------------------

aug3.g20785.t2 VSKLRKPFIKKEWKAHADIAMVNAFFNFFENTIDFPAGILQNAFFNKDRP------------------------------
MSTRG.35640.18 TKKLAK-------------EKVGNF-------------------------------------------------------
MSTRG.35640.16 VSKLRKPFIKKEWKAHADIAMVNAFFNFFENTIDFPAGILQNAFFNKDRP------------------------------
MSTRG.35640.21 VSKLRKPFIKKEWKAHADIAMVNAFFNFFENTIDFPAGILQNAFFNKDRP------------------------------
aug3.g26606.t1 FSKLRKPNVKGDWKDHAGAAVVNAFYNPIENSIEFPAGILQNVFFSNDRP------------------------------
MSTRG.35640.2  VSKLRKPFIKKEWKAHADIAMVNAFFNFFENTIDFPAGILQNAFFNKDRP------------------------------
aug3.g20518.t1 FSKLRKPNVKGE--------------------------------------------------------------------
MSTRG.35640.4  --------------------MVNAFFNFFENTIDFPAGILQNAFFNKDRP------------------------------
MSTRG.35640.6  VSKLRKPFIKKEWKAHADIAMVNAFFNFFENTIDFPAGILQNAFFNKDRP------------------------------
MSTRG.35640.7  --------------------MVNAFFNFFENTIDFPAGILQNAFFNKDRP------------------------------
MSTRG.35640.8  --------------------------------------------------------------------------------
MSTRG.35640.10 --------------------MVNAFFNFFENTIDFPAGILQNAFFNKDRP------------------------------
MSTRG.35640.11 VSKLRKPFIKKEWKAHADIAMVNAFFNFFENTIDFPAGILQNAFFNKDRP------------------------------
aug3.g20517.t1 --------------------------------------------------------------------------------
aug3.g20516.t1 FSKLRKPNIKGE--------------------------------------------------------------------
aug3.g19898.t1 --------------------------------------------------------------------------------
aug3.g19899.t1 FSKLRKPNVKGE--------------------------------------------------------------------
aug3.g19900.t1 --------------------MVNAFFNFFENTIDFPAGILQNAFFNKDRPTYMNFGAIGYVIGHEITHGFDDMGKQFDKE
MSTRG.35640.19 TKKLAK-------------EKVGNF-------------------------------------------------------
aug3.g20786.t1 --------------------------------------------------------------------------------

aug3.g20785.t2 -------------------------------------------TYMNFGAIGYVIGHEITHGFDDMGKQFDKEGNNVNWW
MSTRG.35640.18 --------------------------------------------------------------------------------
MSTRG.35640.16 -------------------------------------------TYMNFGAIGYVIGHEITHGFDDMGKQFDKEGNNVNWW
MSTRG.35640.21 -------------------------------------------TYMNFGAIGYVIGHEITHGFDDMGKQFDKEGNNVNWW
aug3.g26606.t1 -------------------------------------------NYMNYGGIGFVIGHEITHGFDDMGKQFDKDGNNRNWW
MSTRG.35640.2  -------------------------------------------TYMNFGAIGYVIGHEITHGFDDMGKQFDKEGNNVNWW
aug3.g20518.t1 --------------------------------------------------------------------------------
MSTRG.35640.4  -------------------------------------------TYMNFGAIGYVIGHEITHGFDDMGKQFDKEGNNVNWW
MSTRG.35640.6  -------------------------------------------TYMNFGAIGYVIGHEITHGFDDMGKQFDKEGNNVNWW
MSTRG.35640.7  -------------------------------------------TYMNFGAIGYVIGHEITHGFDDMGKQFDKEGNNVNWW
MSTRG.35640.8  --------------------------------------------------------------------------------
MSTRG.35640.10 -------------------------------------------TYMNFGAIGYVIGHEITHGFDDMGKQFDKEGNNVNWW
MSTRG.35640.11 -------------------------------------------TYMNFGAIGYVIGHEITHGFDDMGKQFDKEGNNVNWW
aug3.g20517.t1 ---------------------------------------------MNYGGIGFAIGHEITHGFDDMGKQFDKDGNNRNWW
aug3.g20516.t1 --------------------------------------------------------------------------------
aug3.g19898.t1 --------------------------------------------------------------------------------
aug3.g19899.t1 --------------------------------------------------------------------------------
aug3.g19900.t1 GNNVNWWDQATIDNFNEKANCIIYXXXXXXXXXXXXXXXXXXYSYMNFGAIGYVIGHEITHGFDDMGKQFDKEGNNVNWW
MSTRG.35640.19 --------------------------------------------------------------------------------
aug3.g20786.t1 --------------------------------------------------------------------------------
```

```
aug3.g20785.t2 DQATIDNFNEKANCIIYQYGNYTTENGLSVNGITTQGENIADNGGMKEAYRAYHAWVRDNGPEKKLPGLKYTPSQLFWIS
MSTRG.35640.18 --------------------------------------------------------------------------------
MSTRG.35640.16 DQATIDNFNEKANCIIYQYGNYTTENGLSVNGITTQGENIADNGGMKEAYRAYHAWVRDNGPEKKLPGLKYTPSQLFWIS
MSTRG.35640.21 DQATIDNFNEKANCIIYQYGNYTTENGLSVNGITTQGENIADNGGMKEAYRAYHAWVRDNGPEKKLPGLKYTPSQLFWIS
aug3.g26606.t1 DQETDDNFEEKAKCIIHQYGNYTTEIGLKLNGINTQGENIADNGGIKEAYRGYHSWVKDHGPEGRLPGLRYTPSQLFFIS
MSTRG.35640.2  DQATIDNFNEKANCIIYQYGNYTTENGLSVNGITTQGENIADNGGMKEAYRAYHAWVRDNGPEKKLPGLKYSPSQLFWIS
aug3.g20518.t1 --------------------------------------------------------------------------------
MSTRG.35640.4  DQATIDNFNEKANCIIYQYGNFTAENGLSVNGITTQGENIADNGGMKEAYRAYHAWVRDNGPEKKLPGLKYSPSQLFWIS
MSTRG.35640.6  DQATIDNFNEKANCIIYQYGNFTAENGLSVNGITTQGENIADNGGMKEAYRAYHAWVRDNGPEKKLPGLKYSPSQLFWIS
MSTRG.35640.7  DQATIDNFNEKANCIIYQYGNYTTENGLSVNGITTQGENIADNGGMKEAYRAYHAWVRDNGPEKKLPGLKYTPSQLFWIS
MSTRG.35640.8  --------------------------------------------------------------------------------
MSTRG.35640.10 DQATIDNFNEKANCIIYQYGNYTTENGLSVNGITTQGENIADNGGMKEAYRAYHAWVRDNGPEKKLPGLKYSPSQLFWIS
MSTRG.35640.11 DQATIDNFNEKANCIIYQYGNFTAENGLSVNGITTQGENIADNGGMKEAYRAYHAWVRDNGPEKKLPGLKYSPSQLFWIS
aug3.g20517.t1 DQKTDDNFKEKAKCIIHQYGNYTTEIGLKVNGINTQGENIADNGGIKEAYRGYHSWVKDHGSEGRLPGLRYTPSQLFFIS
aug3.g20516.t1 --------------------------------------------------------------------------------
aug3.g19898.t1 --------------------------------------------------------------------------------
aug3.g19899.t1 --------------------------------------------------------------------------------
aug3.g19900.t1 DQATIDNFNEKANCIIYQYGNFTAENGLSVNGITTQGENIADNGGMKEAYRE----VIDSIDVQTLQRV-----------
MSTRG.35640.19 --------------------------------------------------------------------------------
aug3.g20786.t1 --------------------------------------------------------------------------------

aug3.g20785.t2 SASVWCANYRPEQLKLMILSDPHSPGEFRVNGPMSNLKEFSAAYGCGTETKMNPDHKCQVW-------------------
MSTRG.35640.18 --------------------------------------------------------------------------------
MSTRG.35640.16 SASVWCANYRPEQLKLMILSDPHSPGEFRVNGPMSNLKEFSAAFACGTETKMNPDHKCQVW-------------------
MSTRG.35640.21 SASVWCANYRPEQLKLMILSDPHSPGEFRVNGPMSNLKEFSAAYGCGTETKMNPDHKCQVW-------------------
aug3.g26606.t1 AANVWCSKYRPEQLKLMVLADPHSPGEFRVIGPMSNLKEFSTAFNCPLGSTMNPEKKCEVW-------------------
MSTRG.35640.2  SASVWCANYRPEQLKLMILSDPHSPGEFRVNGPMSNLKEFSAAFGCATETKMNPDHKCQVWNSICGNHHMKFERTLYFFI
aug3.g20518.t1 --------------------------------------------------------------------------------
MSTRG.35640.4  SASVWCANYRPEQLKLMILSDPHSPGEFRVNGPMSNLKEFSAAFGCATETKMNPDHKCQVW-------------------
MSTRG.35640.6  SASVWCANYRPEQLKLMILSDPHSPGEFRVNGPMSNLKEFSAAFGCATETKMNPDHKCQVW-------------------
MSTRG.35640.7  SASVWCANYRPEQLKLMILSDPHSPGEFRVNGPMSNLKEFSAAFGCATETKMNPDHKCQVW-------------------
MSTRG.35640.8  ----------------MILSDPHSPGEFRVNGPMSNLKEFSAAFGCATETKMNPDHKCQVW-------------------
MSTRG.35640.10 SASVWCANYRPEQLKLMILSDPHSPGEFRVNGPMSNLKEFSAAFGCATETKMNPDHKCQVW-------------------
MSTRG.35640.11 SASVWCANYRPEQLKLMILSDPHSPGEFRVNGPMSNLKEFSAAFGCATETKMNPDHKCQVW-------------------
aug3.g20517.t1 AANAWCSKYRPEQLKLMVLVDPHSPGEFRVIGPMSNLKEFSTAFNCPLGSTMNPEKKCEVW-------------------
aug3.g20516.t1 --------------------------------------------------------------------------------
aug3.g19898.t1 ----------------MVLADPHSPGEF----------------------------------------------------
aug3.g19899.t1 --------------------------------------------------------------------------------
aug3.g19900.t1 -----MQDFAIRLYHIITNDDRLIEHVITVNGPMSNLKEFSAAFACGTETKMNPDHKCQVW-------------------
MSTRG.35640.19 --------------------------------------------------------------------------------
aug3.g20786.t1 --------------------------------------------------------------------------------

aug3.g20785.t2 --------------------------------------------------------------------------------
MSTRG.35640.18 --------------------------------------------------------------------------------
MSTRG.35640.16 --------------------------------------------------------------------------------
MSTRG.35640.21 --------------------------------------------------------------------------------
aug3.g26606.t1 --------------------------------------------------------------------------------
MSTRG.35640.2  VTSLLMTLTIPAVFGHNYRKKIDAIQTRFVPLNSPRFVQEANEEAICTTPGCVRTAEEIIQILDEKVNPCDNFYKFACGG
aug3.g20518.t1 --------------------------------------------------------------------------------
MSTRG.35640.4  --------------------------------------------------------------------------------
MSTRG.35640.6  --------------------------------------------------------------------------------
MSTRG.35640.7  --------------------------------------------------------------------------------
MSTRG.35640.8  --------------------------------------------------------------------------------
MSTRG.35640.10 --------------------------------------------------------------------------------
MSTRG.35640.11 --------------------------------------------------------------------------------
aug3.g20517.t1 --------------------------------------------------------------------------------
aug3.g20516.t1 --------------------------------------------------------------------------------
aug3.g19898.t1 --------------------------------------------------------------------------------
aug3.g19899.t1 --------------------------------------------------------------------------------
aug3.g19900.t1 --------------------------------------------------------------------------------
MSTRG.35640.19 --------------------------------------------------------------------------------
aug3.g20786.t1 --------------------------------------------------------------------------------
```

```
aug3.g20785.t2  --------------------------------------------------------------------------------
MSTRG.35640.18  --------------------------------------------------------------------------------
MSTRG.35640.16  --------------------------------------------------------------------------------
MSTRG.35640.21  --------------------------------------------------------------------------------
aug3.g26606.t1  --------------------------------------------------------------------------------
MSTRG.35640.2   WINKHSIPDDKAMMSVFHQVRDVLNLQLKGLLQKPSDESEPKSIKMLDQTSLGMPDRTYLVSGLSDSGTKAYFNLMVKAA
aug3.g20518.t1  --------------------------------------------------------------------------------
MSTRG.35640.4   --------------------------------------------------------------------------------
MSTRG.35640.6   --------------------------------------------------------------------------------
MSTRG.35640.7   --------------------------------------------------------------------------------
MSTRG.35640.8   --------------------------------------------------------------------------------
MSTRG.35640.10  --------------------------------------------------------------------------------
MSTRG.35640.11  --------------------------------------------------------------------------------
aug3.g20517.t1  --------------------------------------------------------------------------------
aug3.g20516.t1  --------------------------------------------------------------------------------
aug3.g19898.t1  --------------------------------------------------------------------------------
aug3.g19899.t1  --------------------------------------------------------------------------------
aug3.g19900.t1  --------------------------------------------------------------------------------
MSTRG.35640.19  --------------------------------------------------------------------------------
aug3.g20786.t1  --------------------------------------------------------------------------------
```

```
aug3.g20785.t2  --------------------------------------------------------------------------------
MSTRG.35640.18  --------------------------------------------------------------------------------
MSTRG.35640.16  --------------------------------------------------------------------------------
MSTRG.35640.21  --------------------------------------------------------------------------------
aug3.g26606.t1  --------------------------------------------------------------------------------
MSTRG.35640.2   KKLGANEETVEKELLEALNFEITLANYSLPREERRDYDSMYHKYTISQLRELVPQIDWLKYLNGLLNDPITETETLIVMV
aug3.g20518.t1  --------------------------------------------------------------------------------
MSTRG.35640.4   --------------------------------------------------------------------------------
MSTRG.35640.6   --------------------------------------------------------------------------------
MSTRG.35640.7   --------------------------------------------------------------------------------
MSTRG.35640.8   --------------------------------------------------------------------------------
MSTRG.35640.10  --------------------------------------------------------------------------------
MSTRG.35640.11  --------------------------------------------------------------------------------
aug3.g20517.t1  --------------------------------------------------------------------------------
aug3.g20516.t1  --------------------------------------------------------------------------------
aug3.g19898.t1  --------------------------------------------------------------------------------
aug3.g19899.t1  --------------------------------------------------------------------------------
aug3.g19900.t1  --------------------------------------------------------------------------------
MSTRG.35640.19  --------------------------------------------------------------------------------
aug3.g20786.t1  --------------------------------------------------------------------------------
```

```
aug3.g20785.t2  --------------------------------------------------------------------------------
MSTRG.35640.18  --------------------------------------------------------------------------------
MSTRG.35640.16  --------------------------------------------------------------------------------
MSTRG.35640.21  --------------------------------------------------------------------------------
aug3.g26606.t1  --------------------------------------------------------------------------------
MSTRG.35640.2   ANFLKQFGELIAKTDKRIVANYMMWRVVADSAGMLSKDWKALVQEYVLAITGEREEKPRWEQCVNSLRENLEIALSSYYI
aug3.g20518.t1  --------------------------------------------------------------------------------
MSTRG.35640.4   --------------------------------------------------------------------------------
MSTRG.35640.6   --------------------------------------------------------------------------------
MSTRG.35640.7   --------------------------------------------------------------------------------
MSTRG.35640.8   --------------------------------------------------------------------------------
MSTRG.35640.10  --------------------------------------------------------------------------------
MSTRG.35640.11  --------------------------------------------------------------------------------
aug3.g20517.t1  --------------------------------------------------------------------------------
aug3.g20516.t1  --------------------------------------------------------------------------------
aug3.g19898.t1  --------------------------------------------------------------------------------
aug3.g19899.t1  --------------------------------------------------------------------------------
aug3.g19900.t1  --------------------------------------------------------------------------------
MSTRG.35640.19  --------------------------------------------------------------------------------
aug3.g20786.t1  --------------------------------------------------------------------------------
```

```
aug3.g20785.t2 --------------------------------------------------------------------------------
MSTRG.35640.18 --------------------------------------------------------------------------------
MSTRG.35640.16 --------------------------------------------------------------------------------
MSTRG.35640.21 --------------------------------------------------------------------------------
aug3.g26606.t1 --------------------------------------------------------------------------------
MSTRG.35640.2  KHYFKGDSKQKASEMVKYISNAFLEILKQIDWMDEETRRQAIEKANAIVSYVGYPQELLDDSLVSQLYENLTITNENYFM
aug3.g20518.t1 --------------------------------------------------------------------------------
MSTRG.35640.4  --------------------------------------------------------------------------------
MSTRG.35640.6  --------------------------------------------------------------------------------
MSTRG.35640.7  --------------------------------------------------------------------------------
MSTRG.35640.8  --------------------------------------------------------------------------------
MSTRG.35640.10 --------------------------------------------------------------------------------
MSTRG.35640.11 --------------------------------------------------------------------------------
aug3.g20517.t1 --------------------------------------------------------------------------------
aug3.g20516.t1 --------------------------------------------------------------------------------
aug3.g19898.t1 --------------------------------------------------------------------------------
aug3.g19899.t1 --------------------------------------------------------------------------------
aug3.g19900.t1 --------------------------------------------------------------------------------
MSTRG.35640.19 --------------------------------------------------------------------------------
aug3.g20786.t1 --------------------------------------------------------------------------------
```

```
aug3.g20785.t2 --------------------------------------------------------------------------------
MSTRG.35640.18 --------------------------------------------------------------------------------
MSTRG.35640.16 --------------------------------------------------------------------------------
MSTRG.35640.21 --------------------------------------------------------------------------------
aug3.g26606.t1 --------------------------------------------------------------------------------
MSTRG.35640.2  NNLNIRKWTTNYYFSKLRKPNVKGDWKDHAGAAVVNAFYNPIENSIEFPAGILQNVFFSNDRPNYMNYGGIGFVIGHEIT
aug3.g20518.t1 --------------------------------------------------------------------------------
MSTRG.35640.4  --------------------------------------------------------------------------------
MSTRG.35640.6  --------------------------------------------------------------------------------
MSTRG.35640.7  --------------------------------------------------------------------------------
MSTRG.35640.8  --------------------------------------------------------------------------------
MSTRG.35640.10 --------------------------------------------------------------------------------
MSTRG.35640.11 --------------------------------------------------------------------------------
aug3.g20517.t1 --------------------------------------------------------------------------------
aug3.g20516.t1 --------------------------------------------------------------------------------
aug3.g19898.t1 --------------------------------------------------------------------------------
aug3.g19899.t1 --------------------------------------------------------------------------------
aug3.g19900.t1 --------------------------------------------------------------------------------
MSTRG.35640.19 --------------------------------------------------------------------------------
aug3.g20786.t1 --------------------------------------------------------------------------------
```

```
aug3.g20785.t2 --------------------------------------------------------------------
MSTRG.35640.18 --------------------------------------------------------------------
MSTRG.35640.16 --------------------------------------------------------------------
MSTRG.35640.21 --------------------------------------------------------------------
aug3.g26606.t1 --------------------------------------------------------------------
MSTRG.35640.2  HGFDDMGKQFDKDGNNRNWWDQETDDNFEEKAKCIIHQYGNYTTEIGLKLNGINTQGENIADNGGIKE
aug3.g20518.t1 --------------------------------------------------------------------
MSTRG.35640.4  --------------------------------------------------------------------
MSTRG.35640.6  --------------------------------------------------------------------
MSTRG.35640.7  --------------------------------------------------------------------
MSTRG.35640.8  --------------------------------------------------------------------
MSTRG.35640.10 --------------------------------------------------------------------
MSTRG.35640.11 --------------------------------------------------------------------
aug3.g20517.t1 --------------------------------------------------------------------
aug3.g20516.t1 --------------------------------------------------------------------
aug3.g19898.t1 --------------------------------------------------------------------
aug3.g19899.t1 --------------------------------------------------------------------
aug3.g19900.t1 --------------------------------------------------------------------
MSTRG.35640.19 --------------------------------------------------------------------
aug3.g20786.t1 --------------------------------------------------------------------
```
