# Supplementary material for: Alternative Transcription at Venom Genes and Its Role as a Complementary Mechanism for the Generation of Venom Complexity in the Common House Spider
Source: Front Ecol Evol. Author manuscript; Available in PMC 2019 Aug 20. (PMC6700725; doi:10.3389/fevo.2019.00085)
Supplement: Data Sheet 8 [file NIHMS1042230-supplement-Data_Sheet_8.PDF]

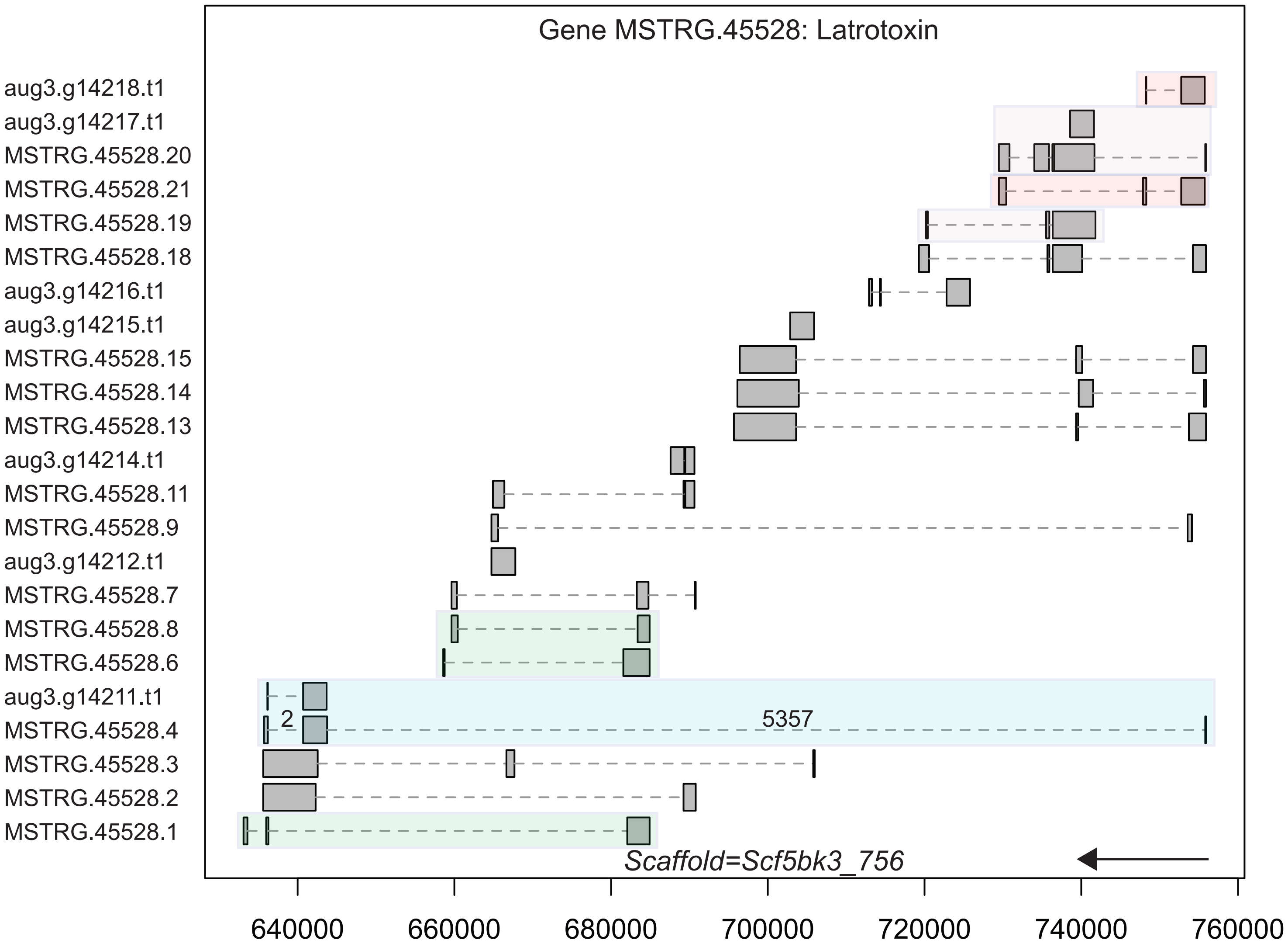

Figure S8. The exon-intron structure of predicted transcripts at gene MSTRG.45528 (latrotoxin) is shown above. The alignment of all distinct proteins predicted at this locus is shown below. Transcripts producing identical proteins are indicated by shaded boxes of the same color. The arrow indicates inferred direction of transcription. Numbers over introns represent spliced read counts for novel junctions across all libraries, where space allows. Values for other novel introns are found in Table S3.

.

```
MSTRG.45528.19 MVLGSDENYSKTGKALMRMTREMSLEQQGANCKKIEKGQNYLDATVAIAGVALGVFALFSMPLLIVVSSA
MSTRG.45528.4  MTLGSDENYSETGKALMRMTREMSLEEQGKNCKKIEKGQNYLDAAVGIAGVALGVFALFSMPLLIVVSSA
MSTRG.45528.21 MVLGSDENYSETGKALMRLTREMTLEEQGANCKKIEKGQNYMDATVAIAGVALGVAALFTMPLLIVVSSS
aug3.g14216.t1 MDLGSNENYLETGKSLMRMTREMSLEEQGANCKTIEKGQNYMDATIGIAGFALGVAALFTMPMLIVVSSA
aug3.g14214.t1 --MGLDSNEDYSGKALMRMTREMTLKQQGENCKKIEKGQNYLTTIVGVAGVALGLATLFAMPLLFVVASA
aug3.g14212.t1 MTLGSDENYSETGKALMRMTREMSLEEQGKNCKKIEKGKNYLDTAVGLAGVALGVFALFSMPLLIVVSSA
aug3.g14215.t1 MDLGSNANYSETGKALMRMTREMSLEEQGANCKKIEKGQNYLDAAIGVAGVALGVFALFTMPLLIVVSSA
MSTRG.45528.1  ----------------------------------------------------------------------
MSTRG.45528.2  ----------------------------------------------------------------------
MSTRG.45528.3  MDLGSNANYSETGKALMRMTREMSLEEQGANCKKIEKGKNYLDTAVGLAGVALGVFALFSMPLLIVVSSA
MSTRG.45528.7  ----------------------------------------------------------------------
MSTRG.45528.9  ----------------------------------------------------------------------
MSTRG.45528.11 --MGLDSNEDYSGKALMRMTREMTLKQQGENCKKIEKGQNYLTTIVGVAGVALGLATLFAMPLLFVVASA
MSTRG.45528.13 MVLGSDENYSETGKALMRLTREMTLEEQGANCKKIEKGQNYMDATVAIAGVALGVAALFTMPLLIVVSSS
MSTRG.45528.14 MVLGSDENYSETGKALMRLTREMTLEEQGANCKKIEKGQNYLDATVAIAGVALGVFALFSMPLLIVVSSA
MSTRG.45528.15 MVLGSDENYSETGKALMRLTREMTLEEQGANCKKIEKGQNYMDATVAIAGVALGVAALFTMPLLIVVSSS
MSTRG.45528.18 MVLGSDENYSETGKALMRLTREMTLEEQGANCKKIEKGQNYMDATVAIAGVALGVAALFTMPLLIVVSSS

MSTRG.45528.19 LLVNVLIAKALTADENIERECADLPFKELDEKMKARQEETDRKIAEQTEILKEVSEKVGQTLDKIEDVRK
MSTRG.45528.4  LLVNVLIAKALTADENIERECADLPFKELDAKMKARQEETDRKIAEQTEILKEVNKKVGQTLDKIEDVRK
MSTRG.45528.21 LLVNVLIAKALTADENIERECADLPFKELDEKMKARQEEQDRKIAEQTEILKEVSEKVGQTLDKIEDVRK
aug3.g14216.t1 LLINVLIAKALTADENIERECADLPFKELDEKMKARQEETDRKIAEQTEILKEVSEKVGQTLDKIEDVRK
aug3.g14214.t1 LLVNVLIAKALTADEKIERECSDVPFKELDEKMKARQEETDRKIAEQTEILKEVNAKVTQTLENIEEARK
aug3.g14212.t1 LLVNVLIAKALVTDENIERECADLPFKELDAKMKARQEETDRKIAEQTEILKEVNKKVGQTLDKIEDVRK
aug3.g14215.t1 ILVNVLIAKALTADENIERECADLPFKELDEKMKARQEETDRKIAEQNEILKEVSEKVGQTLDKIEDVRK
MSTRG.45528.1  ----------------------------------------------------------------------
MSTRG.45528.2  ----------------------------------------------------------------------
MSTRG.45528.3  LLVNVLIAKALVTDENIERECADLPFKELDAKMKARQEETDRKIAEQTEILKEVNKKVGQTLDKIEDVRK
MSTRG.45528.7  ----------------------------------------------------------------------
MSTRG.45528.9  ----------------------------------------------------------------------
MSTRG.45528.11 LLVNVLIAKALTADEKIERECSDVPFKELDEKMKARQEETDRKIAEQTEILKEVNAKVTQTLENIEEARK
MSTRG.45528.13 LLVNVLIAKALTADENIERECADLPFKELDEKMKARQEEQDRKIAEQTEILKEVSEKVGQTLDKIEDVRK
MSTRG.45528.14 LLVNVLIAKALTADENIERECADLPFKELDEKMKARQEETDRKIAEQTEILKEVSEKVGQTLDKIEDVRK
MSTRG.45528.15 LLVNVLIAKALTADENIERECADLPFKELDEKMKARQEEQDRKIAEQTEILKEVSEKVGQTLDKIEDVRK
MSTRG.45528.18 LLVNVLIAKALTADENIERECADLPFKELDEKMKARQEEQDRKIAEQTEILKEVSEKVGQTLDKIEDVRK

MSTRG.45528.19 EMGDSFQRVLNKIGDLDASNPVSEIKTAIESISFGEENKRLATPERYVYIIQQEVAKNSDSSLYNYIGLQ
MSTRG.45528.4  EMGDSFQRVLNKIGDVDSSNPVSEIKTAIESISFGEENKRLATPERYVYIIQQEVATNSDSSLYNYIGLQ
MSTRG.45528.21 EMGDSFQRVLNKIGDVDASNPVSEIKTAIESISFGEENKRLATPERYVYIIQQEVAKNSDSSLYNYIGLQ
aug3.g14216.t1 EMGDNFQRVLNKIGDLDASNPVSEIKTAIESISFSEENKRLATPERYVYIIQQEVAKNSDSSLYNYIGMQ
aug3.g14214.t1 EMSDSFQRVLKEIGDLDASNPVSEIKTAIESISFSEENKRLATPDRYVYIIEQEVATKSDSSLYNHIGLQ
aug3.g14212.t1 EMGDSFQRVLNKIGDVDASNPVSEIKTAIESISFGEENKRLATPERYVYIIQQEVATNSDSSLYNYIGLQ
aug3.g14215.t1 EMGDNFQRVLNKIGDLDASNPVSEIKTAIESISFGEENKRLATPDRYVYIIQQEVAKKSDTSLYNYIGMQ
MSTRG.45528.1  ------------------------------------------------------MGTSIVFFGNRIVILI
MSTRG.45528.2  ----------------------------------------------------------------------
MSTRG.45528.3  EMGDSFQRVLNKIGDVDASNPVSEIKTAIESISFGEENKRLATPERYVYIIQQEVATNSDSSLYNYIGLQ
MSTRG.45528.7  ----------------------------------------------------------------------
MSTRG.45528.9  ----------------------------------------------------------------------
MSTRG.45528.11 EMSDSFQRVLKEIGDLDASNPVSEIKTAIESISFSEENKRLATPDRYVYIIEQEVATKSDSSLYNHIGLQ
MSTRG.45528.13 EMGDSFQRVLNKIGDVDASNPVSEIKTAIESISFGEENKRLATPERYVYIIQQEVAKNSDSSLYNYIGLQ
MSTRG.45528.14 EMGDSFQRVLNKIGDLDASNPVSEIKTAIESISFGEENKRLATPERYVYIIQQEVAKNSDSSLYNYIGLQ
MSTRG.45528.15 EMGDSFQRVLNKIGDVDASNPVSEIKTAIESISFGEENKRLATPERYVYIIQQEVAKNSDSSLYNYIGLQ
MSTRG.45528.18 EMGDSFQRVLNKIGDVDASNPVSEIKTAIESISFGEENKRLATPERYVYIIQQEVAKNSDSSLYNYIGLQ
```

```
MSTRG.45528.19  GSLYKAIFAVINKKNTIKSNIALPGLNIGVTTYASTIITLIQQLRYMSEYVYQKGDLKKFNDYFNRLIFD
MSTRG.45528.4   GSLHKAIFAVINKKNTIKPNIALPGLNIGVTTYASTVITLTQQLRYMSEYVYQKGDLEKFNDYFNRLIFD
MSTRG.45528.21  GSLYKAIFAVINKKNTIKSNIALPGLNIGVTTYASTVITLIQQLRYMSEYVYQKGDLKKFNNYFNRLIFD
aug3.g14216.t1  GSLYNAIFAVINKKNTIKPNIALPGLNIGVTTYASTVITLIQQLRYMSEYAYQKGDLKKFNNYFNRLCFD
aug3.g14214.t1  GSLYKAIFAVINKKNTIKPNIALPGLNIGVTTYASTVITLVQQLRYMSEYVYQKGDLNKFNNYFSRLIFD
aug3.g14212.t1  GSLYKAIFAVINKKNTIKPNIALPGLNIGVTTYASTVITLIQQLRYMSEYVYQKGDLKNFNDYFNRLIFD
aug3.g14215.t1  GSLYKAIFAVINKKNTIKPNIALPGLNIGVTTYASSVITLIQQLRYMSEYVYQKGDLKKFNNFYNRLIFD
MSTRG.45528.1   GSRFKRYFLMFNGIYNFRGQLVLLWMSADTVNFVGKKVRILK-LNKVSRFA--------FNFFFCAYAIN
MSTRG.45528.2   ----------------------------------------------------------------------
MSTRG.45528.3   GSLYKAIFAVINKKNTIKPNIALPGLNIGVTTYASTVITLIQQLRYMSEYVYQKGDLKNFNDYFNRLIFD
MSTRG.45528.7   -------------------------MSADTVNFVGKKVRILK-LNKVSRFA--------FNFFFCAYAIN
MSTRG.45528.9   ----------------------------------------------------------------------
MSTRG.45528.11  GSLYKAIFAVINKKNTIKPNIALPGLNIGVTTYASTVITLVQQLRYMSEYVYQKGDLNKFNNYFSRLIFD
MSTRG.45528.13  GSLYKAIFAVINKKNTIKSNIALPGLNIGVTTYASTVITLIQQLRYMSEYVYQKGDLKKFNNYFNRLIFD
MSTRG.45528.14  GSLYKAIFAVINKKNTIKSNIALPGLNIGVTTYASTIITLIQQLRYMSEYVYQKGDLKKFNDYFNRLIFD
MSTRG.45528.15  GSLYKAIFAVINKKNTIKSNIALPGLNIGVTTYASTVITLIQQLRYMSEYVYQKGDLKKFNNYFNRLIFD
MSTRG.45528.18  GSLYKAIFAVINKKNTIKSNIALPGLNIGVTTYASTVITLIQQLRYMSEYVYQKGDLKKFNNYFNRLIFD


MSTRG.45528.19  FNYFKLIVNGSSKKQGIIDQVTQMLNDAKKSKSKQDLGEELFDNIGT------YITQLNQLKQKIAALSL
MSTRG.45528.4   FNHFKLIVNGSSRKPGIIDQVTQMLNDAKRSKSKQDLGEELFENIGT------YITQLSQLKQKIAALSL
MSTRG.45528.21  FNYFKLIVNGSSKKQGIIDQVTQMLNEAKKSKSKQDLGEELFENIGT------YITQLNQLKQKIAALSL
aug3.g14216.t1  FNYFKLIVNGSSKKQGIIDQVTQMLNEAKKSKSKQDLGDELFDNIGT------YITQLNQLKQKITALSL
aug3.g14214.t1  FNYFKLILNGSSKKQGIIDRVTKILSEAKKNKSKQDLGEELFENIGT------YITQLNQLKQKIAALSL
aug3.g14212.t1  FNHFKLIVNGSSRKPGIIDQVTQMLNDAKRSKSKQDLGEELFENIGT------YINQLNQLKQKIAALSL
aug3.g14215.t1  FNFFKLILNGSSRKQGILDQVIQILNDAKKSKSKQDLGDELFENIGR------YITQLNQLKQKIAALSL
MSTRG.45528.1   FCF-------DSKCLLLSLLILLILCSLQFYKVLADVLLKFHYVLAAIPVCTVFRANRNGAKQLPICFYL
MSTRG.45528.2   ----------------------------------------------------------------------
MSTRG.45528.3   FNHFKLIVNGSSRKPGIIDQVTQMLNDAKRSKSKQDLGEELFENIGT------YINQLNQLKQKIAALSL
MSTRG.45528.7   FCF-------DSKCLLLSLLILLILCSLQFYKVLADVLLKFHYVLAAIPVCTVFRANRNGAKQLPICFYL
MSTRG.45528.9   ----------------------------------------------------------------------
MSTRG.45528.11  FNYFKLILNGSSKKQGIIDRVTKILSEAKKNKSKQDLGEELFENIGT------YITQLNQLKQKIAALSL
MSTRG.45528.13  FNYFKLIVNGSSKKQGIIDQVTQMLNEAKKSKSKQDLGEELFENIGT------YITQLNQLKQKIAALSL
MSTRG.45528.14  FNYFKLIVNGSSKKQGIIDQVTQMLNDAKKSKSKQDLGEELFDNIGT------YITQLNQLKQKIAALSL
MSTRG.45528.15  FNYFKLIVNGSSKKQGIIDQVTQMLNEAKKSKSKQDLGEELFENIGT------YITQLNQLKQKIAALSL
MSTRG.45528.18  FNYFKLIVNGSSKKQGIIDQVTQMLNEAKKSKSKQDLGEELFENIGT------YITQLNQLKQKIAALSL


MSTRG.45528.19  HVLEATPDKNMDIDFNSRTGNQRSSTNFLDWKKGTKVSYAVQFEKDGKYSKVSDWTSPQEIVDIANPDIV
MSTRG.45528.4   PVLETTLDKNIDIDFNSKTGYQRSSSNFLDWKRGTKVSYAMQFEKDGKYSKVSDWTSPQEIVDIANPEIV
MSTRG.45528.21  DVLETTPDKNMDIDFNSRTGNQRSSSNFLDWKKGTRVSYAVQFEKDGKYSKVSGWTSPQEIVDIANPDIV
aug3.g14216.t1  DVLETTPDKNMDIDFNSRTGNQRSSSNFLDWKKGTKVSYAVQFEKDGKYSKVSGWTSPQDIVDIANPDIV
aug3.g14214.t1  DVLETTPDKNMDIDFNSRTGNQRSSSNFLDWKKGTQVSYAVQFEKD------------------------
aug3.g14212.t1  PVLETTPDKNIDIDFNSKTGYQRSSSNFLDWKRGTKVSYAVQFEKDGKYSKVSDWTSPQEIVDIANPEIV
aug3.g14215.t1  NVLESTPDNNLDIDFNSRTGDQLSNTNFLDWKEGTKVSYAVQFEKDGKYSKVSDWTSPQDIMKKANPDIV
MSTRG.45528.1   NILFC---KNVFITSS------------------------------------------------------
MSTRG.45528.2   ----------------------------------------------------------------------
MSTRG.45528.3   PVLETTPDKNIDIDFNSKTGYQRSSSNFLDWKRGTKVSYAMQFEKDGKYSKVSDWTSPQEIVDIANPEIV
MSTRG.45528.7   NILFC---KNVFITSS------------------------------------------------------
MSTRG.45528.9   ----------------------------------------------------------------------
MSTRG.45528.11  DVLETTPDKNMDIDFNSRTGNQRSSSNFLDWKKGTQVSYAVQFEKD------------------------
MSTRG.45528.13  DVLETTPDKNMDIDFNSRTGNQRSSSNFLDWKKGTRVSYAVQFEKDGKYSKVSGWTSPQEIVDIANPDIV
MSTRG.45528.14  HVLEATPDKNMDIDFNSRTGNQRSSTNFLDWKKGTKVSYAVQFEKDGKYSKVSDWTSPQEIVDIANPDIV
MSTRG.45528.15  DVLETTPDKNMDIDFNSRTGNQRSSSNFLDWKKGTRVSYAVQFEKDGKYSKVSGWTSPQEIVDIANPDIV
MSTRG.45528.18  DVLETTPDKNMDIDFNSRTGNQRSSSNFLDWKKGTRVSYAVQFEKDGKYSKVSGWTSPQEIVDIANPDIV
```

```
MSTRG.45528.19 FRKSSNMNRLVFRKFGNGDAELAYILPG-SEVSFRDVHRDLYNLAFKNSLSESQVSSSMDRLIRLGANVK
MSTRG.45528.4  FRKSSNMNRLVFRKFDNGDAELAYILPG-SEVKFRDVHRDLYNLAFKNSLSESQVSSNMDRLIRLGANVK
MSTRG.45528.21 FRKSSNMNRLVFRKFGNGDAELAYILPG-SEVSFRDVHRDLYNLAFKNSLSESQVSSNMDRLIRLGANVK
aug3.g14216.t1 FRKSSNMNRLVFRKFGNGDAELAYILPG-SEVKFRDVHRDLYNLAFKNSLSESQVSSNMDRLIRLGANVK
aug3.g14214.t1 --------------------ELAYILPGGSEVKFRDVHRDLYNLAFKNSLSESQVSSNMVRLIRLGANVK
aug3.g14212.t1 FRKSGNMNRLVFRKFDNGDAELAYILPG-SEVKFRDVHRDLYNLAFKNSLSESQVSSNMDRLIRLGANVK
aug3.g14215.t1 FRKTSNMNRLVFRKFGNGEAELAYILPG-SKSSFRDVHRDLYNLAFKNSLSEAEVESNMDRLIKLGADVK
MSTRG.45528.1  ----------------------------------------------------------------------
MSTRG.45528.2  ----------------------------------------------------------MDRLIRLGANVK
MSTRG.45528.3  FRKSSNMNRLVFRKFDNGDAELAYILPG-SEVKFRDVHRDLYNLAFKNSLSESQVSSNMDRLIRLGANVK
MSTRG.45528.7  ----------------------------------------------------------------------
MSTRG.45528.9  ----------------------------------------------------------------------
MSTRG.45528.11 --------------------ELAYILPGGSEVKFRDVHRDLYNLAFKNSLSESQVSSNMDRLIRLGANVK
MSTRG.45528.13 FRKSSNMNRLVFRKFGNGDAELAYILPG-SEVSFRDVHRDLYNLAFKNSLSESQVSSNMDRLIRLGANVK
MSTRG.45528.14 FRKSSNMNRLVFRKFGNGDAELAYILPG-SEVSFRDVHRDLYNLAFKNSLSESQVSSSMDRLIRLGANVK
MSTRG.45528.15 FRKSSNMNRLVFRKFGNGDAELAYILPG-SEVSFRDVHRDLYNLAFKNSLSESQVSSNMDRLIRLGANVK
MSTRG.45528.18 FRKSSNMNRLVFRKFGNGDAELAYILPG-SEVSFRDVHRDLYNLAFKNSLSESQVSSNMDRLIRLGANVK

MSTRG.45528.19 TVFEGKRTVIHAAAIAGRSVMLGKILEKDRSLINKPDRLGFTPLHLAAENKRTAFARWLINRGANVNLQG
MSTRG.45528.4  AEFEGKRTVIHAAAIAGRSVMLGKILQKDRSLINKPDKFGFTPLHLAAENKRTAFARWLINRGANVNLQG
MSTRG.45528.21 AVFEGKRTVIHAAAIAGRSVMLGKILEKDRSLINKPDKLGFTPLHLAAENKRTAFARWLINRGANVNLQG
aug3.g14216.t1 AVFEGKRTVIHAAAIAGRSVMLGKILEKDRSLINKPDRLGFTPLHLAAENKRTAFARWLINRGANVNLQG
aug3.g14214.t1 AVFEGKRTVIHAAAIAGRSVMLRKILEKDRSLINKPDRLGFTPLHLAAENKRTAFARWLINRGANVNLQG
aug3.g14212.t1 AEFEGKRTVIHAAAIAGRSVMLGKILAKDRSLINKPDKLGFTPLHLAAENKRTAFARWLINRGANVNLQG
aug3.g14215.t1 AVFEGKRTVIHAAAIAGRTVMLGKILEKEGSLINLQDKLGFTPLHLAAENKRTDFAQGLINRGANVNVQG
MSTRG.45528.1  ----------------------------------------------------------------------
MSTRG.45528.2  AEFEGKRTVIHAAAIAGRSVMLGKILQKDRSLINKPDKFGFTPLHLAAENKRTAFARWLINRGANVNLQG
MSTRG.45528.3  AEFEGKRTVIHAAAIAGRSVMLGKILQKDRSLINKPDKFGFTPLHLAAENKRTAFARWLINRGANVNLQG
MSTRG.45528.7  ----------------------------------------------------------------------
MSTRG.45528.9  ----------------------------------------------------------------------
MSTRG.45528.11 AEFEGKRTVIHAAAIAGRSVMLGKILAKDRSLINKPDKLGFTPLHLAAENKRTAFARWLINRGANVNLQG
MSTRG.45528.13 AVFEGKRTVIHAAAIAGRSVMLGKILEKDRSLINKPDKLGFTPLHLAAENKRTAFARWLINRGANVNLQG
MSTRG.45528.14 TVFEGKRTVIHAAAIAGRSVMLGKILEKDRSLINKPDRLGFTPLHLAAENKRTAFARWLINRGANVNLQG
MSTRG.45528.15 AVFEGKRTVIHAAAIAGRSVMLGKILEKDRSLINKPDRLGFTPLHLAAENKRTAFARWLINRGANVNLQG
MSTRG.45528.18 AVFEGKRTVIHAAAIAGRSVMLGKILEKDRSLINKPDRLGFTPLHLAAENKRTAFARWLINRGANVNLQG

MSTRG.45528.19 QEYKVTPLHLAVRYHAEEIVEALLDKSNINPNLKDVAGLTPLHYAVTDENYNKHLFTTLIKSRKTDLNVK
MSTRG.45528.4  QEYKVTPLHLAVRYHAEEIVEDLLRNSKIDPNLKDIAGLTPLHYAITDENYNKDLFLTLIENGKTNLNAK
MSTRG.45528.21 QEYKVTPLHLAVRYHAEEIVEALLDKSNINPNLKDVAGLTPLHYAVTDENLNKEIFTSLKLSRKTDFNVK
aug3.g14216.t1 QEFKVSPLHLAVRYHADEIVEDLLESSKINPNLKDIAGLTPLHYAVTDENYDKYFFTTLIDNEKTDLNVK
aug3.g14214.t1 QEYKITPLHLAVRYHADEIVEDLLENSKIDPNLKDVAGLTPLHYAVTDENYNKELFLTLIRNRKTNLNAK
aug3.g14212.t1 QEYKVTPLHLAVRYQAEEIVEDLLRNSKIDPNLKDIAGLTPLHYALTDEIYNKELFLTLMRNGKTNLNAK
aug3.g14215.t1 QEYKVSPLHLAVRYHAEEIVKALLENSNINPNLKDIAGLTPLHYAVTDENYNKKLFTTLLTNRKTDLNVK
MSTRG.45528.1  ----------------------------------------------------------------------
MSTRG.45528.2  QEYKVTPLHLAVRYHAEEIVEDLLRNSKIDPNLKDIAGLTPLHYAITDENYNKDLFLTLIENGKTNLNAK
MSTRG.45528.3  QEYKVTPLHLAVRYHAEEIVEDLLRNSKIDPNLKDIAGLTPLHYAITDENYNKDLFLTLIENGKTNLNAK
MSTRG.45528.7  ----------------------------------------------------------------------
MSTRG.45528.9  ----------------------------------------------------------------------
MSTRG.45528.11 QEYKVTPLHLAVRYQAEEIVEDLLRNSKIDPNLKDIAGLTPLHYALTDEIYNKELFLTLMRNGKTNLNAK
MSTRG.45528.13 QEYKVTPLHLAVRYHAEEIVEALLDKSNINPNLKDVAGLTPLHYAVTDENLNKEIFTSLKLSRKTDFNVK
MSTRG.45528.14 QEYKVTPLHLAVRYHAEEIVEALLDKSNINPNLKDVAGLTPLHYAVTDENYNKHLFTTLIKSRKTDLNVK
MSTRG.45528.15 QEYKVTPLHLAVRYHAEEIVEALLDKSNINPNLKDVAGLTPLHYAVTDENYNKHLFTTLIKSRKTDLNVK
MSTRG.45528.18 QEYKVTPLHLAVRYHAEEIVEALLDKSNINPNLKDVAGLTPLHYAVTDENYNKHLFTTLIKSRKTDLNVK
```

```
MSTRG.45528.19 DNNGLAVVHYATILDRWEEVIDLTIEGDRFDIYAKDNQQMLAVHYDAMKGNKNEQLISSMMADDKASKLN
MSTRG.45528.4  DNNGLAVVHYATILDRWEEVTDLSTKDKRFDIYAKDNQQMLAVHYDAMKGNKVEQLISSIMFDEKASKLN
MSTRG.45528.21 DNNGLPVLHYATILNRWEEVTDLIFTAERCDYYAKDNQQMLAVHYDAMKGNKVDILISAMMGDEKASKLN
aug3.g14216.t1 DNNGLAVVHYATILNRWEEVIDLTYKKERFDIFAKDNQQMLAVHYDAMKGNQVEELVA--MEDDKASKLN
aug3.g14214.t1 DNNGLAVVHYATILNRWEEVIDLAIQNNRFDIYAKDNQQMLAVHYDAMKGNKVEKLISSLMFDEKASKLN
aug3.g14212.t1 DNNGLAVVHYATILDRWEEVIDLSTQDNRFDIYAKDNQQMLAVHYDAMKGNKVEQLISSIMLDKKASKLN
aug3.g14215.t1 DNNGLAVVHYATILNRWEEVVDLTFEGDRFDIYAKDNQQMLAVHYDAMKGYKNEQLISSMMADDKASKLN
MSTRG.45528.1  ----------------------------------------------------------------------
MSTRG.45528.2  DNNGLAVVHYATILDRWEEVTDLSTKDKRFDIYAKDNQQMLAVHYDAMKGNKVEQLISSIMFDEKASKLN
MSTRG.45528.3  DNNGLAVVHYATILDRWEEVTDLSTKDKRFDIYAKDNQQMLAVHYDAMKGNKVEQLISSIMFDEKASKLN
MSTRG.45528.7  ----------------------------------------------------------------------
MSTRG.45528.9  ---------------------------------------MLAVHYDAMKGNKVDILISAMMGDEKASKLN
MSTRG.45528.11 DNNGLAVVHYATILDRWEEVIDLSTQDNRFDIYAKDNQQMLAVHYDAMKGNKVEQLISSIMLDKKASKLN
MSTRG.45528.13 DNNGLPVLHYATILNRWEEVTDLIFTAERCDYYAKDNQQMLAVHYDAMKGNKNEQLISSMMADDKASKLN
MSTRG.45528.14 DNNGLAVVHYATILDRWEEVIDLTIEGDRFDIYAKDNQQMLAVHYDAMKGYKNEQLISSMMADDKASKLN
MSTRG.45528.15 DNNGLAVVHYATILDRWEEVIDLTIEGDRFDIYAKDNQQMLAVHYDAMKGNKNEQLISSMMADDKASKLN
MSTRG.45528.18 DNNGLAVVHYATILDRWEEVIDLTIEGDRFDIYAKDNQQMLAVHYDAMKGNKNEQLISSMMADDKASKLN

MSTRG.45528.19 DAAGEKHWTPLHYAVFFKQTAWVKYLFHWHERVISKINVDAKDVDNQTPLHLAAAAGLKDIVKILLEKGA
MSTRG.45528.4  DAAGEKHWTPLHFAVFFKQTAWVQYLLNFHENVVSKVNVDAKDVDNQTPLHLAAAAGLKEIVKELLAKGA
MSTRG.45528.21 DAAGEKHWTPLHYAVYFKHTDWVEYLFRQHERVVSKVNVDAKDVDDQTPLHLAAGAGLKDIVKTLLDNGA
aug3.g14216.t1 DAAGEKHWTPLHYAVFFKQTAWVKYLFRQQKRVVSKINVDAKDVDDQTPLHLAAAAGLKDIVKTLLEKGA
aug3.g14214.t1 DAAGEKHWTPLHFAVFFKQTAWVEYLFGQHENVVSKVNVDAKDVDNQTPLHLAAGAGLKDIVKILLDNGA
aug3.g14212.t1 DAAGEKHWTPLHFAVFFKQTAWVEYLFGQHKRVVSKVNVDAKDVDNQTPLHLAAAAGLKDIVKILLDNGA
aug3.g14215.t1 DAAGEKHWTPLHYAAFFKQTAWVKYLFHWHENVVSKINVDAKDVDDQTPLHLAAAAGLKEIVKELLANGA
MSTRG.45528.1  ----------------------------------------------------------------------
MSTRG.45528.2  DAAGEKHWTPLHFAVFFKQTAWVQYLLNFHENVVSKVNVDAKDVDNQTPLHLAAAAGLKEIVKELLAKGA
MSTRG.45528.3  DAAGEKHWTPLHFAVFFKQTAWVQYLLNFHENVVSKVNVDAKDVDNQTPLHLAAAAGLKEIVKELLAKGA
MSTRG.45528.7  ----------------------------------------------------------------------
MSTRG.45528.9  DAAGEKHWTPLHYAVYFKHTDWVEYLFRQHERVVSKVNVDAKDVDNQTPLHLAAAAGLKDIVKILLDNGA
MSTRG.45528.11 DAAGEKHWTPLHFAVFFKQTAWVEYLFGQHKRVVSKVNVDAKDVDNQTPLHLAAAAGLKDIVKILLDNGA
MSTRG.45528.13 DAAGEKHWTPLHYAVFFKQTAWVKYLFHWHERVISKINVDAKDVDNQTPLHLAAAAGLKDIVKILLEKGA
MSTRG.45528.14 DAAGEKHWTPLHYAAFFKQTAWVKYLFHWHENVVSKINVDAKDVDDQTPLHLAAAAGLKEIVKELLANGA
MSTRG.45528.15 DAAGEKHWTPLHYAVFFKQTAWVKYLFHWHERVISKINVDAKDVDNQTPLHLAAAAGLKDIVKILLEKGA
MSTRG.45528.18 DAAGEKHWTPLHYAVFFKQTAWVKYLFHWHERVISKINVDAKDVDNQTPLHLAAAAGLKDIVKILLEKGA

MSTRG.45528.19 KVYEKTKK-QNTPLDLAVMHNRKEVIDDLLTFEKNQKLKNGKSAKENDSKACQLAKGEMLNLLKKKNRCG
MSTRG.45528.4  KVYEKTKK-QNTPLDLAVMHNRKEVIDDLLTFEKNQKLKNGKSAKENDAKACQLAKGEMLNLLKKKNRCG
MSTRG.45528.21 KVYEKTKK-QNTPLDLAVMHNRKEVIDDLLTFEKNQKLKNGKSAKENDTKACQLAKGEMLNLLKKKNRCG
aug3.g14216.t1 KVYEKTKK-QNTPLDLAVMHNRKEVIDDLLTFEKDQKLKNGKPAKENDAKACQLAKGEMLDLLKKMDRCG
aug3.g14214.t1 KVYEKTKK-QNAPLDLAVMHNRKEVIDDLLTFEKNQKLNNGKSAKENDTKACQLAKGEMLNLLKKKNRCG
aug3.g14212.t1 KVYEKTKK-QNTPLDLAVMHNRKEVIDDLLTFEKNQKLKNGKSAKENDTKACQLAKGEMLNLLKKKNRCG
aug3.g14215.t1 KVYEKTKK-QNTPLDLAVMHNRKEVIDDLLTFENNQKLKNGKSAKENDTKACQLAKGEMLNLLKKKNRCG
MSTRG.45528.1  ----------------------------------------------------------------------
MSTRG.45528.2  KVYEKTKK-QNTPLDLAVMHNRKEVIDDLLTFEKNQKLKNGKSAKENDAKACQLAKGEMLNLLKKKNRCG
MSTRG.45528.3  KVYEKTKK-QNTPLDLAVMHNRKEVIDDLLTFEKNQKLKNGKSAKENDAKACQLAKGEMLNLLKKKNRCG
MSTRG.45528.7  ----------------------------------------------------------------------
MSTRG.45528.9  KVYEKTKK-QNTPLDLAVMHNRKEVIDDLLTFEKNQKLKNGKSAKENDTKACQLAKGEMLNLLKKKNRCG
MSTRG.45528.11 KVYEKTKK-QNTPLDLAVMHNRKEVIDDLLTFEKNQKLKNGKSAKENDTKACQLAKGEMLNLLKKKNRCG
MSTRG.45528.13 KVLRKDEETKYTS---------------------------------------------------------
MSTRG.45528.14 KVYEKTKK-QNTPLDLAVMHNRKEVIDDLLTFENNQKLKNGKSAKENDTKACQLAKGEMLNLLKKKNRCG
MSTRG.45528.15 KVLRKDEETKYTS---------------------------------------------------------
MSTRG.45528.18 KVYEKTKK-QNTPLDLAVMHNRKEVIDDLLTFEKNQKLKNGKSAKENDSKACQLAKGEMLNLLKKKNRCG
```

```
MSTRG.45528.19 NFRRSIESNSKNSARGFTLTGMFENNNLRPLLISNKPKENLEFKNTKPLTQVDVNGALLLLDLFVRKVTN
MSTRG.45528.4  NFRRSIESNSENPAPEFTLTGMLENSNLRPLLNSNKLKENLELKNIKPLTQMDVNGALLLLDLFVRKVTN
MSTRG.45528.21 NFRRSVESNSKTSAWGFTLTGMFENNNLRPLLTSNKSRENLEFKNIKPLTQVDVNGALLLLDLFVRKVTN
aug3.g14216.t1 NFRRSIESNSKNSSREITLTGMFENNNLRPLLTSNKPKENLAFKNIKPLTQVDVNGALLLLDLFVRKVTN
aug3.g14214.t1 NFRRSIKSNSKNSARGFTLTGMLENNSLRPLLISNKPKENLEFKNIKPLTQVDVNGALLLLDLFVRKVTN
aug3.g14212.t1 NFRRSIESNFQNSARESSLMGMFENNNLRPLLNSNKPKENLEFKNIKPLTQMDVNGALLLLDLFVRKVTN
aug3.g14215.t1 NFRRSNDDNSTNSPRKFAPIGMSENNSLRPLLISNKPKENLELKNINPLTQVDVNGALLLLDLFIRKMTN
MSTRG.45528.1  ----------------------------------------------------------------------
MSTRG.45528.2  NFRRSIESNSENPAPEFTLTGMLENSNLRPLLNSNKLKENLELKNIKPLTQMDVNGALLLLDLFVRKVTN
MSTRG.45528.3  NFRRSIESNSENPAPEFTLTGMLENSNLRPLLNSNKLKENLELKNIKPLTQMDVNGALLLLDLFVRKVTN
MSTRG.45528.7  ----------------------------------------------------------------------
MSTRG.45528.9  NFRRSIESNFQNSARESSLMGMFENNNLRPLLNSNKPKENLEFKNIKPLTQMDVNGALLLLDLFVRKVTN
MSTRG.45528.11 NFRRSIESNFQNSARESSLMGMFENNNLRPLLNSNKPKENLEFKNIKPLTQMDVNGALLLLDLFVRKVTN
MSTRG.45528.13 ----------------------------------------------------------------------
MSTRG.45528.14 NFRRSNDDNSTNSPRKFAPIGMSENNSLRPLLISNKPKENLELKNINPLTQVDVNGALLLLDLFIRKMTN
MSTRG.45528.15 ----------------------------------------------------------------------
MSTRG.45528.18 NFRRSIESNSKNSARGFTLTGMFENNNLRPLLISNKPKENLEFKNTKPLTQVDVNGALLLLDLFVRKVTN

MSTRG.45528.19 EKYNSAVIGSESMLDARARALNITDNLQKIMEMTGNDGDLFDIYSKIYKAIRSGNDSKLGKEVCIYLNQY
MSTRG.45528.4  EKYNSALIGSESLLDARARALNITDNLQKIMEMAGNGGDLFDIHSKIYKAIMRGNDSKLVKEVCTYLNEY
MSTRG.45528.21 EKYNSAVIGSESLLDARARALNITENLQKIVEMAGNDGDLFDIHSKIYKAIMSGNDYKLVKEVCTYLKEY
aug3.g14216.t1 EKYNSAVIGSESLLDARARALNVTGNLQKIMEMAGNDGDLFDIHSKIYKAIMSGNDSNLVKEVCTYLNRY
aug3.g14214.t1 EKYNSAVIGSESLLDARALALNITDNLQKIMEMAGNDGDLFGIHSKIYKAFMSGNDYKLVKEICTYLNKY
aug3.g14212.t1 EKYNSAVIGSESLLDARARALNITDNLQKIVEMAGNDGDLFDIHSKIYKAIMSGNDSKLVKEVCTYLNEF
aug3.g14215.t1 EKYNSAWIGSQSLLDARARALNITDNLQKIMEIAGNDGDLFDIHSKIYKAIMSGNDYKLVKEVCTYLNQY
MSTRG.45528.1  ----------------------------------------------------------------------
MSTRG.45528.2  EKYNSALIGSESLLDARARALNITDNLQKIMEMAGNGGDLFDIHSKIYKAIMRGNDSKLVKEVCTYLNEY
MSTRG.45528.3  EKYNSALIGSESLLDARARALNITDNLQKIMEMAGNGGDLFDIHSKIYKAIMRGNDSKLVKEVCTYLNEY
MSTRG.45528.7  ----------------------------------------------------------------------
MSTRG.45528.9  EKYNSAVIGSESLLDARARALNITDNLQKIVEMAGNDGDLFDIHSKIYKAIMSGNDSKLVKEVCTYLNEF
MSTRG.45528.11 EKYNSAVIGSESLLDARARALNITDNLQKIVEMAGNDGDLFDIHSKIYKAI-------------------
MSTRG.45528.13 ----------------------------------------------------------------------
MSTRG.45528.14 EKYNSAWIGSQSLLDARARALNITDNLQKIMEIAGNDGDLFDIHSKIYKAIMSGNDYKLVKEVCTYLNQY
MSTRG.45528.15 ----------------------------------------------------------------------
MSTRG.45528.18 EKYNSAVIGSESMLDARARALNITDNLQKIMEMTGNDGDLFDIYSKIYKAIRSGNDSKLGKEVCIYLNQY

MSTRG.45528.19 STLEPQEVENFISAMVEKKSVGITKKAI-P-SNLE-NLVAQACYRNLF----------------------
MSTRG.45528.4  STLEPQKVENFISGMVENKSVKISKKVI-QVRPTT-SLFSYYIRIIRYNIATE-----------------
MSTRG.45528.21 STLEPHKVENFISAMVENKSVGITKKAIQE-SFID-SMLKVG----------------------------
aug3.g14216.t1 STLEPQKVEKFISAMFENKSARITRKAIQESGQLSVGLVSQVGPFNQILRSFSVDGGTPLTSLSVGLSYP
aug3.g14214.t1 STLEPQEVEKFIFAMVENKSAEITKKII-Q-GNFE-NLVAQACYRNHY----------------------
aug3.g14212.t1 STLEPQKVENFISGMVENKSVGISKKVI-Q-GNYK-NLVKQACYSNHY----------------------
aug3.g14215.t1 STLEPQKVENIISAMVENKSFGITKNAI-Q-GNLE-NLIAQACYRNNF----------------------
MSTRG.45528.1  ----------------------------------------------------------------------
MSTRG.45528.2  STLEPQKVENFISGMVENKSVKISKKVI-Q-GNFE-NLVAHICFQNHY----------------------
MSTRG.45528.3  STLEPQKVENFISGMVENKSVKISKKVI-Q-GNFE-NLVAHICFQNHY----------------------
MSTRG.45528.7  ----------------------------------------------------------------------
MSTRG.45528.9  STLEPQKVENFISGMVENKSVGISKKVI-Q-GNYK-NLVKQACYSNHY----------------------
MSTRG.45528.11 ----------------------------------------------------------------------
MSTRG.45528.13 ----------------------------------------------------------------------
MSTRG.45528.14 STLEPQKVENIISAMVENKSFGITKNAI-Q-GNLE-NLIAQACYRNNF----------------------
MSTRG.45528.15 ----------------------------------------------------------------------
MSTRG.45528.18 STLEPQEVENFISAMVEKKSVGITKKAI-P-SNLE-NLVAQACYRNLF----------------------
```

```
MSTRG.45528.19 ----------------------------------------------------------------------
MSTRG.45528.4  ----------------------------------------------------------------------
MSTRG.45528.21 ----------------------------------------------------------------------
aug3.g14216.t1 GQCRNCEGGAVGPEFIFMDDNARPHRALMVGEYLEGEDIQRMDWPAKSPDLNPIEHVWDALGRAIAMHQP
aug3.g14214.t1 ----------------------------------------------------------------------
aug3.g14212.t1 ----------------------------------------------------------------------
aug3.g14215.t1 ----------------------------------------------------------------------
MSTRG.45528.1  ----------------------------------------------------------------------
MSTRG.45528.2  ----------------------------------------------------------------------
MSTRG.45528.3  ----------------------------------------------------------------------
MSTRG.45528.7  ----------------------------------------------------------------------
MSTRG.45528.9  ----------------------------------------------------------------------
MSTRG.45528.11 ----------------------------------------------------------------------
MSTRG.45528.13 ----------------------------------------------------------------------
MSTRG.45528.14 ----------------------------------------------------------------------
MSTRG.45528.15 ----------------------------------------------------------------------
MSTRG.45528.18 ----------------------------------------------------------------------
```

```
MSTRG.45528.19 ----------------------------------------------------------------
MSTRG.45528.4  ----------------------------------------------------------------
MSTRG.45528.21 ----------------------------------------------------------------
aug3.g14216.t1 PPRTYLELKISLVEEWEDLPQVFLNSLINSMHTRCACCLSGVTIHHTRGNTHCASLTFIVIFSP
aug3.g14214.t1 ----------------------------------------------------------------
aug3.g14212.t1 ----------------------------------------------------------------
aug3.g14215.t1 ----------------------------------------------------------------
MSTRG.45528.1  ----------------------------------------------------------------
MSTRG.45528.2  ----------------------------------------------------------------
MSTRG.45528.3  ----------------------------------------------------------------
MSTRG.45528.7  ----------------------------------------------------------------
MSTRG.45528.9  ----------------------------------------------------------------
MSTRG.45528.11 ----------------------------------------------------------------
MSTRG.45528.13 ----------------------------------------------------------------
MSTRG.45528.14 ----------------------------------------------------------------
MSTRG.45528.15 ----------------------------------------------------------------
MSTRG.45528.18 ----------------------------------------------------------------
```
