# Supplementary material for: Alternative Transcription at Venom Genes and Its Role as a Complementary Mechanism for the Generation of Venom Complexity in the Common House Spider
Source: Front Ecol Evol. Author manuscript; Available in PMC 2019 Aug 20. (PMC6700725; doi:10.3389/fevo.2019.00085)
Supplement: Data Sheet 9 [file NIHMS1042230-supplement-Data_Sheet_9.PDF]

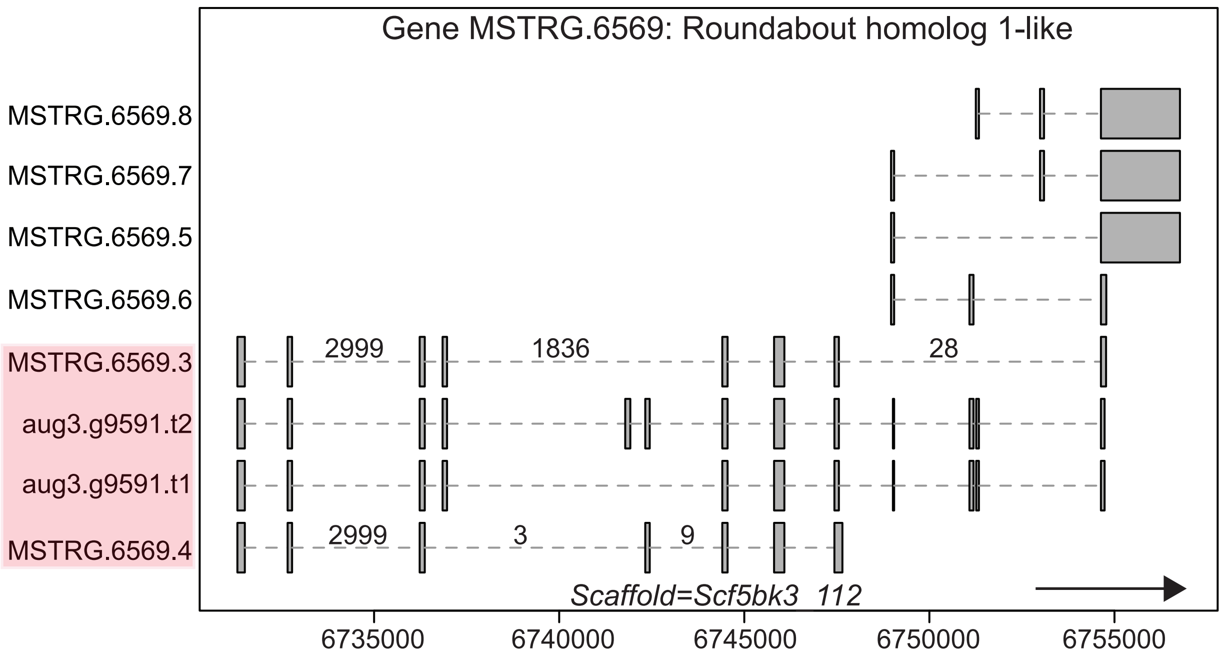


```
               ********************************************************************************
MSTRG.6569.3   MLFAGQMKITKDDRINVDGTSLVISNVHPKDSGEYTCQISTIPPLELSHTLDVLYPPTVKPHPKDGFINVKEGETVSLSC
MSTRG.6569.4   MLFAGQMKITKDDRINVDGTSLVISNVHPKDSGEYTCQISTIPPLELSHTLDVLYPPTVKPHPKDGFINVKEGETVSLSC
aug3.g9591.t1  MLFAGQMKITKDDRINVDGTSLVISNVHPKDSGEYTCQISTIPPLELSHTLDVLYPPTVKPHPKDGFINVKEGETVSLSC
aug3.g9591.t2  MLFAGQMKITKDDRINVDGTSLVISNVHPKDSGEYTCQISTIPPLELSHTLDVLYPPTVKPHPKDGFINVKEGETVSLSC

               **********************************************************
MSTRG.6569.3   AASGNPKPIIRWKHAGVHYDSSDDTNFRINSAQKSDSGEYECIADNGVGDPASAVITV----------------------
MSTRG.6569.4   AASGNPKPIIRWKHAGVHYDSSDDTNFRINSAQKSDSGEYECIADNGVGDPASAVITV----------------------
aug3.g9591.t1  AASGNPKPIIRWKHAGVHYDSSDDTNFRINSAQKSDSGEYECIADNGVGDPASAVITV----------------------
aug3.g9591.t2  AASGNPKPIIRWKHAGVHYDSSDDTNFRINSAQKSDSGEYECIADNGVGDPASAVITVEVVSAPKVYPNPRSGLLTVKEG

                                                                                ***************
MSTRG.6569.3   -----------------------------------------------------------------EVVSAPKVYPNPRSG
MSTRG.6569.4   -----------------------------------------------------------------EVVSAPKVYPNPRSG
aug3.g9591.t1  -----------------------------------------------------------------EVVSAPKVYPNPRSG
aug3.g9591.t2  ETISISCEATGDPVPVITWKRPGVHYDSSDDTNFRINSAQKSDSGEYECIADNGVGDPASAVITVEVVSAPKVYPNPRSG

               ********************************************************************************
MSTRG.6569.3   LLTVKEGETISISCEATGDPVPVITWKRPGGEKATKVREENKIVISEANRSHAGSYECIANNSLGIFTSAIIRVQVQYAP
MSTRG.6569.4   LLTVKEGETISISCEATGDPVPVITWKRPGGEKATKVREENKIVISEANRSHAGSYECIANNSLGIFTSAIIRVQVQYAP
aug3.g9591.t1  LLTVKEGETISISCEATGDPVPVITWKRPGGEKATKVREENKIVISEANRSHAGSYECIANNSLGIFTSAIIRVQVQYAP
aug3.g9591.t2  LLTVKEGETISISCEATGDPVPVITWKRPGGEKATKVREENKIVISEANRSHAGSYECIANNSLGIFTSAIIRVQVQYAP

               ********************************************************************************
MSTRG.6569.3   EITVPHSWIHAGEGTNVNITCIIRGEPIPSVMWKKGGDGQVIHGNDHYHMKTDGHLHMLEIRHLKEDDFGRYDCIASNSL
MSTRG.6569.4   EITVPHSWIHAGEGTNVNITCIIRGEPIPSVMWKKGGDGQVIHGNDHYHMKTDGHLHMLEIRHLKEDDFGRYDCIASNSL
aug3.g9591.t1  EITVPHSWIHAGEGTNVNITCIIRGEPIPSVMWKKGGDGQVIHGNDHYHMKTDGHLHMLEIRHLKEDDFGRYDCIASNSL
aug3.g9591.t2  EITVPHSWIHAGEGTNVNITCIIRGEPIPSVMWKKGGDGQVIHGNDHYHMKTDGHLHMLEIRHLKEDDFGRYDCIASNSL

               ***************************************************
MSTRG.6569.3   SSVSENIHIIGTPQVPHFTSKPHISQGTKYTISWIVDSHTPVDEYSLMFRK-----------------------------
MSTRG.6569.4   SSVSENIHIIGTPQVPHFTSKPHISQGTKYTISWIVDSHTPVDEYSLMFRK-----------------------------
aug3.g9591.t1  SSVSENIHIIGTPQVPHFTSKPHISQGTKYTISWIVDSHTPVDEYSLMFRKSEAFVFTTKKEEPSTSSAQASSTSAGAIP
aug3.g9591.t2  SSVSENIHIIGTPQVPHFTSKPHISQGTKYTISWIVDSHTPVDEYSLMFRKSEAFVFTTKKEEPSTSSAQASSTSAGAIP

                             :
MSTRG.6569.3   --------------INIQKVLSAAR---------------------------------------LLPM----------
MSTRG.6569.4   --------------VSSV------------------------------------------------------------
aug3.g9591.t1  AEGTTEIPIDIGNDIPIINAVVTARNKFGWSKQSEAFVFTTKKEDKHSESALSSSSTTYVNLMFTIPMVFVSLYRNLL
aug3.g9591.t2  AEGTTEIPIDIGNDIPIINAVVTARNKFGWSKQSEAFVFTTKKEDKHSESALSSSSTTYVNLMFTIPMVFVSLYRNLL
```

Figure S9. The exon-intron structure of predicted transcripts at gene MSTRG.6569 (roundabout homolog 1-like) is shown at top.The bottom four transcripts, whose names are shaded pink, produce non-identical proteins with venom identification by shared peptides only. The alignment of these four distinct proteins is shown at bottom, with the position of the two shared peptides identified in the MS experiment shown by colored bars above and below the protein sequences. The arrow indicates inferred direction of transcription. Numbers over introns represent spliced read counts for novel junctions across all libraries, where space allows. Values for other novel introns are found in Table S3.
